# Impact of postoperative acute kidney injury in patients undergoing major gastrointestinal surgery on 1-year survival and renal outcomes: a national multicentre cohort study

**DOI:** 10.1093/bjsopen/zrab134

**Published:** 2022-01-14

**Authors:** STARSurg Collaborative, Kenneth A McLean, Sivesh K Kamarajah, Emily Mills, James C Glasbey, Dmitri Nepogodiev, James C Glasbey, Aditya Borakati, Joshua Burke, Thomas M Drake, Sivesh K Kamarajah, Kenneth A McLean, Michael F Bath, Henry A Claireaux, Buket Gundogan, Midhun Mohan, Praveena Deekonda, Chia Kong, Holly Joyce, Lisa Mcnamee, Evelina Woin, Chetan Khatri, J Edward Fitzgerald, Ewen M Harrison, Aneel Bhangu, Dmitri Nepogodiev, Nishkantha Arulkumaran, Samira Bell, Fiona Duthie, Jeremy Hughes, Thomas D Pinkney, John Prowle, Toby Richards, Mark Thomas, K Dynes, M Patel, P Patel, C Wigley, R Suresh, A Shaw, S Klimach, P Jull, D Evans, R Preece, I Ibrahim, V Manikavasagar, R Smith, F S Brown, P Deekonda, R Teo, D P Y Sim, A Borakati, A E Logan, I Barai, H Amin, S Suresh, R Sethi, W Gul, W Bolton, O Corbridge, L Horne, M Attalla, R Morley, C Robinson, T Hoskins, R McAllister, S Lee, Y Dennis, G Nixon, E Heywood, H Wilson, L Ng, S Samaraweera, A Mills, C Doherty, E Woin, J Belchos, V Phan, M Arnold, S Sheik-Ali, R Suresh, A Cordaro, E Mills, N Lorch, D Thomas, B Ibrahim, S Chee, T Ngan, S Pronin, N Thakral, T Yeoh, J Wilson, R Goodson, P Molloy, M Akhbari, W Gul, R Helliwell, S Rees, M Al-Attar, N Griffiths, J Mayes, P Thomas, S George, A Thind, M Kerr, F Shafiq, I Yasin, M Gallagher, E Sewart, T Chouari, T Gardner, N Goergen, J D B Hayes, C S MacLeod, R McCormack, A McKinley, S McKinstry, W Milligan, L Ooi, N M Rafiq, T Sammut, E Sinclair, M Smith, C Baker, A P R Boulton, J Collins, H C Copley, N Fearnhead, H Fox, T Mah, J McKenna, V Naruka, N Nigam, B Nourallah, S Perera, A Qureshi, S Saggar, L Sun, X Wang, D D Yang, P Caroll, C Doyle, S Elangovan, A Falamarzi, K Gascon Perai, E Greenan, D Jain, M Lang-Orsini, S Lim, L O'Byrne, P Ridgway, S Van der Laan, J Wong, J Arthur, J Barclay, P Bradley, C Edwin, E Finch, E Hayashi, M Hopkins, D Kelly, M Kelly, N McCartan, A Ormrod, A Pakenham, J Hayward, C Hitchen, A Kishore, T Martins, J Philomen, R Rao, C Rickards, N Burns, M Copeland, C Durand, A Dyal, A Ghaffar, A Gidwani, M Grant, C Gribbon, A Gruhn, M Leer, K Ahmad, G Beattie, M Beatty, G Campbell, G Donaldson, S Graham, D Holmes, S Kanabar, H Liu, C McCann, R Stewart, S Vara, O Ajibola-Taylor, E J E Andah, C Ani, N M O Cabdi, G Ito, M Jones, A Komoriyama, P Patel, L Titu, M Basra, P Gallogly, G Harinath, S H Leong, A Pradhan, I Siddiqui, S Zaat, A Ali, M Galea, W L Looi, J C K Ng, G Atkin, A Azizi, Z Cargill, Z China, J Elliot, R Jebakumar, J Lam, G Mudalige, C Onyerindu, M Renju, V Shankar Babu, M Hussain, N Joji, B Lovett, H Mownah, B Ali, B Cresswell, A K Dhillon, Y S Dupaguntla, C Hungwe, J D Lowe-Zinola, J C H Tsang, K Bevan, C Cardus, A Duggal, S Hossain, M McHugh, M Scott, F Chan, R Evans, E Gurung, B Haughey, B Jacob-Ramsdale, M Kerr, J Lee, E McCann, K O'Boyle, N Reid, F Hayat, S Hodgson, R Johnston, W Jones, M Khan, T Linn, S Long, P Seetharam, S Shaman, B Smart, A Anilkumar, J Davies, J Griffith, B Hughes, Y Islam, D Kidanu, N Mushaini, I Qamar, H Robinson, M Schramm, C Yan Tan, H Apperley, C Billyard, J M Blazeby, S P Cannon, S Carse, A Göpfert, A Loizidou, J Parkin, E Sanders, S Sharma, G Slade, R Telfer, I Whybrow Huppatz, E Worley, L Chandramoorthy, C Friend, L Harris, P Jain, M J Karim, K Killington, J McGillicuddy, C Rafferty, N Rahunathan, T Rayne, Y Varathan, N Verma, D Zanichelli, M Arneill, F Brown, B Campbell, L Crozier, J Henry, C McCusker, P Prabakaran, R Wilson, U Asif, M Connor, S Dindyal, N Math, A Pagarkar, H Saleem, I Seth, S Sharma, N Standfield, T Swartbol, R Adamson, J E Choi, O El Tokhy, W Ho, N R Javaid, M Kelly, A S Mehdi, D Menon, I Plumptre, S Sturrock, J Turner, O Warren, E Crane, B Ferris, C Gadsby, J Smallwood, M Vipond, V Wilson, T Amarnath, A Doshi, C Gregory, K Kandiah, B Powell, H Spoor, C Toh, R Vizor, M Common, K Dunleavy, S Harris, C Luo, Z Mesbah, A Prem Kumar, A Redmond, S Skulsky, T Walsh, D Daly, L Deery, E Epanomeritakis, M Harty, D Kane, K Khan, R Mackey, J McConville, K McGinnity, G Nixon, A Ang, J Y Kee, E Leung, S Norman, S V Palaniappan, P Partha Sarathy, T Yeoh, J Frost, P Hazeldine, L Jones, M Karbowiak, C Macdonald, A Mutarambirwa, A Omotade, M Runkel, G Ryan, N Sawers, C Searle, S Suresh, S Vig, A Ahmad, R McGartland, R Sim, A Song, J Wayman, R Brown, L H Chang, K Concannon, C Crilly, T J Arnold, A Burgin, F Cadden, C H Choy, M Coleman, D Lim, J Luk, P Mahankali-Rao, A J Prudence-Taylor, D Ramakrishnan, J Russell, A Fawole, J Gohil, B Green, A Hussain, L McMenamin, L McMenamin, M Tang, F Azmi, S Benchetrit, T Cope, A Haque, A Harlinska, R Holdsworth, T Ivo, J Martin, T Nisar, A Patel, K Sasapu, J Trevett, G Vernet, A Aamir, C Bird, A Durham-Hall, W Gibson, J Hartley, N May, V Maynard, S Johnson, C McDonald Wood, M O'Brien, J Orbell, T D Stringfellow, F Tenters, S Tresidder, W Cheung, A Grant, N Tod, M Bews-Hair, Z H Lim, S W Lim, M Vella-Baldacchino, S Auckburally, A Chopada, S Easdon, R Goodson, F McCurdie, M Narouz, A Radford, E Rea, O Taylor, T Yu, M Alfa-Wali, L Amani, I Auluck, P Bruce, J Emberton, R Kumar, N Lagzouli, A Mehta, A Murtaza, M Raja, I S Dennahy, K Frew, A Given, Y Y He, M A Karim, E MacDonald, E McDonald, D McVinnie, S K Ng, A Pettit, D P Y Sim, S D Berthaume-Hawkins, R Charnley, K Fenton, D Jones, C Murphy, J Q Ng, R Reehal, H Robinson, S S Seraj, E Shang, A Tonks, P White, A Yeo, P Chong, R Gabriel, N Patel, E Richardson, L Symons, D Aubrey-Jones, S Dawood, M Dobrzynska, S Faulkner, H Griffiths, F Mahmood, P Patel, M Perry, A Power, R Simpson, A Ali, P Brobbey, A Burrows, P Elder, R Ganyani, C Horseman, P Hurst, H Mann, K Marimuthu, S McBride, E Pilsworth, N Powers, P Stanier, R Innes, T Kersey, M Kopczynska, N Langasco, N Patel, R Rajagopal, B Atkins, W Beasley, Z Cheng Lim, A Gill, H Li Ang, H Williams, T Yogeswara, R Carter, M Fam, J Fong, J Latter, M Long, S Mackinnon, C McKenzie, J Osmanska, V Raghuvir, A Shafi, K Tsang, L Walker, K Bountra, O Coldicutt, D Fletcher, S Hudson, S Iqbal, T Lopez Bernal, J W B Martin, F Moss-Lawton, J Smallwood, M Vipond, A Cardwell, K Edgerton, J Laws, A Rai, K Robinson, K Waite, J Ward, H Youssef, C Knight, P Y Koo, A Lazarou, S Stanger, C Thorn, M C Triniman, A Botha, L Boyles, S Cumming, S Deepak, A Ezzat, A J Fowler, A M Gwozdz, S F Hussain, S Khan, H Li, B Lu Morrell, J Neville, R Nitiahpapand, O Pickering, H Sagoo, E Sharma, K Welsh, S Denley, S Khan, M Agarwal, N Al-Saadi, R Bhambra, A Gupta, Z A R Jawad, L R Jiao, K Khan, G Mahir, S Singagireson, B L Thoms, B Tseu, R Wei, N Yang, N Britton, D Leinhardt, M Mahfooz, A Palkhi, M Price, S Sheikh, M Barker, D Bowley, M Cant, U Datta, M Farooqi, A Lee, G Morley, M Naushad Amin, A Parry, S Patel, S Strang, N Yoganayagam, A Adlan, S Chandramoorthy, Y Choudhary, K Das, M Feldman, B France, R Grace, H Puddy, P Soor, M Ali, P Dhillon, A Faraj, L Gerard, M Glover, H Imran, S Kim, Y Patrick, J Peto, A Prabhudesai, R Smith, A Tang, N Vadgama, R Dhaliwal, T Ecclestone, A Harris, D Ong, D Patel, C Philp, E Stewart, L Wang, E Wong, Y Xu, T Ashaye, T Fozard, F Galloway, S Kaptanis, P Mistry, T Nguyen, F Olagbaiye, M Osman, Z Philip, R Rembacken, S Tayeh, K Theodoropoulou, A Herman, J Lau, A Saha, M Trotter, O Adeleye, D Cave, T Gunwa, J Magalhães, S Makwana, R Mason, M Parish, H Regan, P Renwick, G Roberts, D Salekin, C Sivakumar, A Tariq, I Liew, A McDade, D Stewart, M Hague, N Hudson-Peacock, C E S Jackson, F James, J Pitt, E Y Walker, R Aftab, J J Ang, S Anwar, J Battle, E Budd, J Chui, H Crook, P Davies, S Easby, E Hackney, B Ho, S Z Imam, J Rammell, H Andrews, C Perry, P Schinle, P Ahmed, T Aquilina, E Balai, M Church, E Cumber, A Curtis, G Davies, Y Dennis, E Dumann, S Greenhalgh, P Kim, S King, K H M Metcalfe, L Passby, N Redgrave, Z Soonawalla, S Waters, A Zornoza, I Gulzar, J Hole, K Hull, H Ishaq, J Karaj, A Kelkar, E Love, S Patel, D Thakrar, M Vine, A Waterman, N P Dib, N Francis, M Hanson, R Ingleton, K S Sadanand, N Sukirthan, S Arnell, M Ball, N Bassam, G Beghal, A Chang, V Dawe, A George, T Huq, A Hussain, B Ikram, L Kanapeckaite, M Khan, D Ramjas, A Rushd, S Sait, M Serry, E Yardimci, S Capella, L Chenciner, C Episkopos, E Karam, C McCarthy, W Moore-Kelly, N Watson, V Ahluwalia, J Barnfield, O Ben-Gal, I Bloom, A Gharatya, K Khodatars, N Merchant, A Moonan, M Moore, K Patel, H Spiers, K Sundaram, J Turner, M F Bath, J Black, H Chadwick, L Huisman, H Ingram, S Khan, L Martin, M Metcalfe, P Sangal, J Seehra, A Thatcher, S Venturini, I Whitcroft, Z Afzal, S Brown, A Gani, A Gomaa, N Hussein, S Y Oh, N Pazhaniappan, E Sharkey, T Sivagnanasithiyar, C Williams, J Yeung, L Cruddas, S Gurjar, A Pau, R Prakash, R Randhawa, L Chen, I Eiben, M Naylor, D Osei-Bordom, R Trenear, J Bannard-Smith, N Griffiths, B Y Patel, F Saeed, H Abdikadir, M Bennett, R Church, S E Clements, J Court, A Delvi, J Hubert, B Macdonald, F Mansour, R R Patel, R Perris, S Small, A Betts, N Brown, A Chong, C Croitoru, A Grey, P Hickland, C Ho, D Hollington, L McKie, A R Nelson, H Stewart, P Eiben, M Nedham, I Ali, T Brown, S Cumming, C Hunt, C Joyner, C McAlinden, J Roberts, D Rogers, A Thachettu, N Tyson, R Vaughan, N Verma, T Yasin, K Andrew, N Bhamra, S Leong, R Mistry, H Noble, F Rashed, N R Walker, L Watson, M Worsfold, E Yarham, H Abdikadir, A Arshad, B Barmayehvar, L Cato, N Chan-lam, V Do, A Leong, Z Sheikh, T Zheleniakova, J Coppel, S T Hussain, R Mahmood, R Nourzaie, J Prowle, S Sheik-Ali, A Thomas, A Alagappan, R Ashour, H Bains, J Diamond, J Gordon, B Ibrahim, M Khalil, D Mittapalli, Y N Neo, P Patil, F S Peck, N Reza, I Swan, M Whyte, S Chaudhry, J Hernon, H Khawar, J O'Brien, M Pullinger, K Rothnie, S Ujjal, S Bhatte, J Curtis, S Green, A Mayer, G Watkinson, K Chapple, T Hawthorne, M Khaliq, L Majkowski, T A M Malik, K Mclauchlan, B Ng Wei En, T O'Connor, S Parton, S D Robinson, M I Saat, B N Shurovi, K Varatharasasingam, A E Ward, K Behranwala, M Bertelli, J Cohen, F Duff, O Fafemi, R Gupta, M Manimaran, J Mayhew, D Peprah, M H Y Wong, N Farmer, C Houghton, N Kandhari, K Khan, D Ladha, J Mayes, F McLennan, P Panahi, H Seehra, R Agrawal, I Ahmed, S Ali, F Birkinshaw, M Choudhry, S Gokani, S Harrogate, S Jamal, F Nawrozzadeh, A Swaray, A Szczap, J Warusavitarne, M Abdalla, N Asemota, R Cullum, M Hartley, C Maxwell-Armstrong, C Mulvenna, J Phillips, A Yule, L Ahmed, K D Clement, N Craig, E Elseedawy, D Gorman, L Kane, J Livie, V Livie, E Moss, A Naasan, F Ravi, P Shields, Y Zhu, M Archer, H Cobley, R Dennis, C Downes, B Guevel, E Lamptey, H Murray, A Radhakrishnan, S Saravanabavan, M Sardar, C Shaw, V Tilliridou, R Wright, W Ye, N Alturki, R Helliwell, E Jones, D Kelly, S Lambotharan, K Scott, R Sivakumar, L Victor, H Boraluwe-Rallage, P Froggatt, S Haynes, Y M A Hung, A Keyte, L Matthews, E Evans, P Haray, I John, A Mathivanan, L Morgan, O Oji, C Okorocha, A Rutherford, H Spiers, N Stageman, A Tsui, R Whitham, A Amoah-Arko, E Cecil, A Dietrich, H Fitzpatrick, C Guy, J Hair, J Hilton, L Jawad, E McAleer, Z Taylor, J Yap, M Akhbari, D Debnath, T Dhir, M Elbuzidi, M Elsaddig, S Glace, H Khawaja, R Koshy, K Lal, L Lobo, A McDermott, J Meredith, M A Qamar, A Vaidya, F Acquaah, L Barfi, N Carter, D Gnanappiragasam, C Ji, F Kaminski, S Lawday, K Mackay, S K Sulaiman, R Webb, P Ananthavarathan, F Dalal, E Farrar, R Hashemi, M Hossain, J Jiang, M Kiandee, J Lex, L Mason, J H Matthews, E McGeorge, S Modhwadia, T Pinkney, A Radotra, L Rickard, L Rodman, A Sales, K L Tan, A Bachi, D S Bajwa, J Battle, L R Brown, A Butler, A Calciu, E Davies, I Gardner, T Girdlestone, O Ikogho, G Keelan, P O'Loughlin, J Tam, J Elias, M Ngaage, J Thompson, S Bristow, E Brock, H Davis, M Pantelidou, A Sathiyakeerthy, K Singh, A Chaudhry, G Dickson, P Glen, K Gregoriou, H Hamid, A Mclean, P Mehtaji, G Neophytou, S Potts, D R Belgaid, J Burke, J Durno, N Ghailan, M Hanson, V Henshaw, U R Nazir, I Omar, B J Riley, J Roberts, G Smart, K Van Winsen, A Bhatti, M Chan, M D'Auria, S Green, C Keshvala, H Li, C Maxwell-Armstrong, M Michaelidou, L Simmonds, C Smith, A Wimalathasan, J Abbas, C Cairns, Y R Chin, A Connelly, S Moug, A Nair, D Svolkinas, P Coe, D Subar, H Wang, V Zaver, J Brayley, P Cookson, L Cunningham, A Gaukroger, M Ho, A Hough, J King, D O'Hagan, A Widdison, R Brown, B Brown, A Chavan, S Francis, L Hare, J Lund, N Malone, B Mavi, A McIlwaine, S Rangarajan, N Abuhussein, H S Campbell, J Daniels, I Fitzgerald, S Mansfield, A Pendrill, D Robertson, Y W Smart, T Teng, J Yates, A Belgaumkar, A Katira, J Kossoff, S Kukran, C Laing, B Mathew, T Mohamed, S Myers, R Novell, B L Phillips, M Thomas, T Turlejski, S Turner, M Varcada, L Warren, W Wynell-Mayow, R Church, L Linley-Adams, G Osborn, M Saunders, R Spencer, M Srikanthan, S Tailor, A Tullett, M Ali, S Al-Masri, G Carr, O Ebhogiaye, S Heng, S Manivannan, J Manley, L E McMillan, C Peat, B Phillips, S Thomas, H Whewell, G Williams, A Bienias, E A Cope, G R Courquin, L Day, C Garner, A Gimson, C Harris, K Markham, T Moore, T Nadin, C Phillips, S M Subratty, K Brown, J Dada, M Durbacz, T Filipescu, E Harrison, E D Kennedy, E Khoo, D Kremel, I Lyell, S Pronin, R Tummon, C Ventre, L Walls, E Wootton, A Akhtar, E Davies, D El-Sawy, M Farooq, M Gaddah, H Griffiths, I Katsaiti, N Khadem, K Leong, I Williams, C S Chean, D Chudek, H Desai, N Ellerby, A Hammad, S Malla, B Murphy, O Oshin, P Popova, S Rana, T Ward, T E F Abbott, O Akpenyi, F Edozie, R El Matary, W English, S Jeyabaladevan, C Morgan, V Naidu, K Nicholls, S Peroos, J Prowle, S Sansome, H D Torrance, D Townsend, J Brecher, H Fung, Z Kazmi, P Outlaw, K Pursnani, N Ramanujam, A Razaq, M Sattar, S Sukumar, T S E Tan, K Chohan, S Dhuna, T Haq, S Kirby, J Lacy-Colson, P Logan, Q Malik, J McCann, Z Mughal, S Sadiq, I Sharif, C Shingles, A Simon, S Burnage, S S N Chan, A R J Craig, J Duffield, A Dutta, M Eastwood, F Iqbal, F Mahmood, W Mahmood, C Patel, A Qadeer, A Robinson, A Rotundo, A Schade, R D Slade, M De Freitas, H Kinnersley, E McDowell, S Moens-Lecumberri, J Ramsden, T Rockall, L Wiffen, S Wright, C Bruce, V Francois, K Hamdan, C Limb, A J Lunt, L Manley, M Marks, C F E Phillips, C J F Agnew, C J Barr, N Benons, S J Hart, D Kandage, R Krysztopik, P Mahalingam, J Mock, S Rajendran, M T Stoddart, B Clements, H Gillespie, S Lee, R McDougall, C Murray, R O'Loane, S Periketi, S Tan, R Amoah, R Bhudia, B Dudley, A Gilbert, B Griffiths, H Khan, N McKigney, B Roberts, R Samuel, A Seelarbokus, A Stubbing-Moore, G Thompson, P Williams, N Ahmed, R Akhtar, E Chandler, I Chappelow, H Gil, T Gower, A Kale, G Lingam, L Rutler, C Sellahewa, A Sheikh, H Stringer, R Taylor, H Aglan, M R Ashraf, S Choo, E Das, J Epstein, R Gentry, D Mills, Y Poolovadoo, N Ward, K Bull, A Cole, J Hack, S Khawari, C Lake, T Mandishona, R Perry, S Sleight, S Sultan, T Thornton, S Williams, T Arif, A Castle, P Chauhan, R Chesner, T Eilon, S Kamarajah, C Kambasha, L Lock, T Loka, F Mohammad, S Motahariasl, L Roper, S S Sadhra, A Sheikh, T Toma, Q Wadood, J Yip, E Ainger, S Busti, L Cunliffe, T Flamini, S Gaffing, C Moorcroft, M Peter, L Simpson, E Stokes, G Stott, J Wilson, J York, A Yousaf, A Borakati, M Brown, A Goaman, B Hodgson, A Ijeomah, U Iroegbu, G Kaur, C Lowe, S Mahmood, Z Sattar, P Sen, A Szuman, N Abbas, M Al-Ausi, N Anto, R Bhome, L Eccles, J Elliott, E J Hughes, A Jones, A S Karunatilleke, J S Knight, C C F Manson, I Mekhail, L Michaels, T M Noton, E Okenyi, T Reeves, I H Yasin, D A Banfield, R Harris, D Lim, C Mason-Apps, T Roe, J Sandhu, N Shafiq, E Stickler, J P Tam, L M Williams, P Ainsworth, Y Boualbanat, C Doull, E Egan, L Evans, K Hassanin, G Ninkovic-Hall, W Odunlami, M Shergill, M Traish, D Cummings, S Kershaw, J Ong, F Reid, H Toellner, A Alwandi, M Amer, D George, K Haynes, K Hughes, L Peakall, Y Premakumar, N Punjabi, A Ramwell, H Sawkins, J Ashwood, A Baker, C Baron, I Bhide, E Blake, C De Cates, R Esmail, H Hosamuddin, J Kapp, N Nguru, M Raja, F Thomson, H Ahmed, G Aishwarya, R Al-Huneidi, S Ali, R Aziz, D Burke, B Clarke, A Kausar, D Maskill, L Mecia, L Myers, A C D Smith, G Walker, N Wroe, C Donohoe, D Gibbons, P Jordan, C Keogh, A Kiely, P Lalor, M McCrohan, C Powell, M Power Foley, J Reynolds, E Silke, O Thorpe, J Tseun Han Kong, C White, Q Ali, J Dalrymple, Y Ge, H Khan, R S Luo, H Paine, B Paraskeva, L Parker, K Pillai, J Salciccioli, S Selvadurai, V Sonagara, L R Springford, L Tan, S Appleton, N Leadholm, Y Zhang, D Ahern, M Cotter, S Cremen, T Durrigan, V Flack, N Hrvacic, H Jones, B Jong, K Keane, P R O'Connell, J O'sullivan, G Pek, S Shirazi, C Barker, A Brown, W Carr, Y Chen, C Guillotte, J Harte, A Kokayi, K Lau, S McFarlane, S Morrison, J Broad, N Kenefick, D Makanji, V Printz, R Saito, O Thomas, H Breen, S Kirk, C H Kong, A O'Kane, M Eddama, A Engledow, S K Freeman, A Frost, C Goh, G Lee, R Poonawala, A Suri, P Taribagil, H Brown, S Christie, S Dean, R Gravell, E Haywood, F Holt, E Pilsworth, R Rabiu, H W Roscoe, S Shergill, A Sriram, A Sureshkumar, L C Tan, A Tanna, A Vakharia, S Bhullar, S Brannick, E Dunne, M Frere, M Kerin, K Muthu Kumar, T Pratumsuwan, R Quek, M Salman, N Van Den Berg, C Wong, J Ahluwalia, R Bagga, C M Borg, C Calabria, A Draper, M Farwana, H Joyce, A Khan, M Mazza, G Pankin, M S Sait, N Sandhu, N Virani, J Wong, K Woodhams, N Croghan, S Ghag, G Hogg, O Ismail, N John, K Nadeem, M Naqi, S M Noe, A Sharma, S Tan, F Begum, R Best, A Collishaw, J Glasbey, D Golding, B Gwilym, P Harrison, T Jackman, N Lewis, Y L Luk, T Porter, S Potluri, M Stechman, S Tate, D Thomas, B Walford, F Auld, A Bleakley, S Johnston, C Jones, J Khaw, S Milne, S O'Neill, K K R Singh, R Smith, A Swan, N Thorley, S Yalamarthi, Z D Yin, A Ali, V Balian, R Bana, K Clark, C Livesey, G McLachlan, M Mohammad, N Pranesh, C Richards, F Ross, M Sajid, M Brooke, J Francombe, J Gresly, S Hutchinson, K Kerrigan, E Matthews, S Nur, L Parsons, A Sandhu, M Vyas, F White, A Zulkifli, L Zuzarte, A Al-Mousawi, J Arya, S Azam, A Azri Yahaya, K Gill, R Hallan, C Hathaway, I Leptidis, L McDonagh, S Mitrasinovic, N Mushtaq, N Pang, G B Peiris, S Rinkoff, L Chan, E Christopher, M M H Farhan-Alanie, A Gonzalez-Ciscar, C J Graham, H Lim, K A McLean, H M Paterson, A Rogers, C Roy, D Rutherford, F Smith, G Zubikarai, R Al-Khudairi, M Bamford, M Chang, J Cheng, C Hedley, R Joseph, B Mitchell, S Perera, L Rothwell, A Siddiqui, J Smith, K Taylor, O Wroe Wright, H K Baryan, G Boyd, H Conchie, L Cox, J Davies, S Gardner, N Hill, K Krishna, F Lakin, S Scotcher, J Alberts, M Asad, J Barraclough, A Campbell, D Marshall, W Wakeford, P Cronbach, F D'Souza, E Gammeri, J Houlton, M Hall, A Kethees, R Patel, M Perera, J Prowle, M Shaid, E Webb, S Beattie, M Chadwick, O El-Taji, S Haddad, M Mann, M Patel, K Popat, L Rimmer, H Riyat, H Smith, C Anandarajah, M Cipparrone, K Desai, C Gao, E T Goh, M Howlader, N Jeffreys, A Karmarkar, G Mathew, H Mukhtar, E Ozcan, A Renukanthan, N Sarens, C Sinha, A Woolley, R Bogle, O Komolafe, F Loo, D Waugh, R Zeng, A Crewe, J Mathias, A Mills, A Owen, A Prior, I Saunders, A Baker, L Crilly, J McKeon, H K Ubhi, A Adeogun, R Carr, C Davison, S Devalia, A Hayat, R B Karsan, C Osborne, K Scott, C Weegenaar, M Wijeyaratne, F Babatunde, E Barnor-Ahiaku, G Beattie, P Chitsabesan, O Dixon, N Hall, N Ilenkovan, T Mackrell, N Nithianandasivam, J Orr, F Palazzo, M Saad, L Sandland-Taylor, J Sherlock, T Ashdown, S Chandler, T Garsaa, J Lloyd, S Y Loh, S Ng, C Perkins, A Powell-Chandler, F Smith, R Underhill, N Goergen, A McKinley, C Neary, N Rafiq, A Badran, N Fearnhead, M Leadon, M Yin Lin Ting, K Conlon, D Ganesan, D O'Connor, M J Arthur, Z Panayi, S Rehman, H Awni, R Rao, A Robinson, J Baxter, P Loughlin, A Ahmed, H Barrow, M T Liviu, G Harinath, S Raveendran, S Sait, A Ali, M Latter, S Udalov, M Bergstrom, H Tabry, E West, S Dindyal, C Gao, H Patel, M Bath, K Bevan, M Bica, X M Chan, J Lee, S O'Donnell, M Ravindran, E Blessing, J H De Sousa Magalhaes, P Jain, B Campbell, R Evans, S Poo, C Sanghera, N Standfield, D Karponis, A Mehdi, R Patel, O Warren, G Boyd, J O'Callaghan, M Vipond, T Amarnath, A Kumar, M Saat, S Davidson, A Hylands, E McKie, R Hughes, J Latter, E Leung, P Dos Santos Jorge, J Saramunda, S Vig, P Serebriakoff, J Wayman, S K Yen, M Coleman, S Leong, I Sajid, T Tolppa, A Fawole, D Kandola, A Khan, F Babatunde, A Harlinska, K Sasapu, A D Durham-Hall, G Fowler, M Glithero, J Orbell, T Stringfellow, A Tulloch, A Bagchi, A Grant, O Onibere, M Bews-Hair, N Rajaraman, T Agarwal, S Rabinowicz, A Radford, E Pedlar, A Raja, H Rshaidat, P Y A Aw, E MacKle, E Y L Yap, R Charnley, L A M Lim, M Naylor, B Stainer, N Alseed, R Amarasinghe, R Rajagopal, P Horgan, S Sohrabi, A Wilkinson, N Liew, J Smallwood, M Vipond, N Walker, E Mutengesa, T Rankin, K Waite, E Robertson-Waters, S Stanger, C Thorn, A Botha, A Fowler, T Suri, P Vickers, S Denley, W Johnston, L Jiao, A Pain, K Vutipongsatorn, A Kale, R S Karri, K Waite, C Johnson, J Smith, C Walsh, N Dewan, J Prowle, K Theodoropoulou, P Jain, T Nisar, A Ali, L Chung, J Thomas, M Abbas, S Mookerjee, J Pitt, E Budd, T Fung, M Li, D MacAfee, N Havers, A Kelkar, M Hanson, R Ingleton, N Sukirthan, A Chang, I Eiben, M Qamar, H Javanmard, N Watson, D Bahadori, I Bloom, G Pike, J Black, M Metcalfe, A Radhakrishnan, J Seehra, K Almeida, H Amin, R Holdsworth, J Yeung, S Gurjar, R Jones, M Patel, A Alam, H Ali, J BannardSmith, R Khaw, A Rais, R Ahluwalia, E Briggs, H Gil, J Clements, R Cowden, L McCarthy, N Bassam, S Chan, S F Hussain, R Hryniv, H Noble, J Olivier, J Coppel, J Prowle, S Sait, E Elseedawy, A Hassane, I Ibrahim, T Melaugh, A Ali, L Ashraf, S Green, K Chapple, E Heywood, N Ngonyamo, I Nyamali, A Patil, T Bamford, O Fafemi, C Grieco, K Khan, A Martin, H Seehra, A Burke-Smith, N Johnson, G Samarth, K Sun, J Warusavitarne, S Green, C Maxwell-Armstrong, J Sivaraj, A Campbell, M Elseedawy, E Elseedawy, O Kouli, S Bradbury, R Dennis, H Walji, J Hale, P Haray, P Eiben, A Light, T Singhal, N Carter, F Ewbank, C Perrott, I Chappelow, R Hashemi, A Lee, J Matthews, T Pinkney, M Byrne, H Eltyeb, P O'Loughlin, C Donaldson, O Oke, K Bisset, P Glen, S Norman, L Tan, M Ahmed, C Maxwell-Armstrong, S Rangarajan, J Sivaraj, C Hancock, S Moug, S Smith, G Nowell, B Rigney, A Widdinson, C Boereboom, J Lund, W Simpson, J Wright, I Fitzgerald, S Mansfield, E Shakweh, K Whitehurst, Z Lee, B Pinnell, G Williams, R Broll, T Drake, E Harrison, C McCann, T Abbott, S Mahdi, F Nawab, J Prowle, B Butcher, P D Loganathan, L A Paterson, K Pursnani, J Atley, K Hamdan, E Mills, B Clements, G Donaldson, L Eaton, R Aftab, M Gough, B Griffiths, C Ng, G Nolan, J Archer, V Do, S Sharma, J Epstein, P Sodde, B J Storey, H Ahmad, N Akram, T Sami, F Sheldon, H Croft, L Han, K Lasithiotakis, J Acharya, O Adeleye, G Kaur, N Dabab, P Kangesu, J Knight, K Srikathrikamanathan, H Wilson, E Dell, L Ellis, K McDonald, D Sobhanpanah, K Foster, J Mogg, S Subramonia, P Hill, A Rahem, F Reid, R Bachar, N Greenough, L Hlukha, A Ramwell, S Carlton-Carew, M Murray, A Raja, D Burke, M El-Haddad, L B Mecia, N Patel, R Bhatt, W J Koay, L Y H Low, J Reynolds, S Abbott, H Devan Nair, J J Lee, R O'Connell, W Carr, S Davies, S Unsworth, J Ashcroft, D Lazenby, D Subar, S Choi, S Rinkoff, N Sarens, M Varcada, N Ellerby, A Hammad, N McCartan, U Muhammad, M Howlader, E Norman, P Polly, S Brown, T Clark, N Thakral, P Hann, R Henderson, S Kirk, S Gupta, T Richards, J Ting, M Byrne, C Byrne, J Cheema, S Walsh, C Borg, J Hardie, Y Sardar, B Hughes, S Saeed, F Saeed, A Sharma, E Ang, B Kansu, M Stechman, R Walford, C Woodward, S Adeyemi, R Awad, L Imam, I Leptidis, E D Kennedy, H Patterson, Z M Soh, L Walls, J D Yau, B Ali, D Evans, J Smith, E James, V E Kantola, K Krishna, H Naeem, J Prowle, O Komolafe, E Tilling, C Osborne, J Schuster Bruce, C Weegenaar, P Chitsabesan, A Goaman, C Goode, N Nithianandavisam

## Abstract

**Background:**

The intermediate-term impact of acute kidney injury (AKI) in patients after major gastrointestinal and liver surgery has not been well characterized. This study aimed to evaluate the 1-year mortality rate and renal outcomes associated with postoperative AKI in a national prospective cohort.

**Methods:**

This prospective multicentre, observational cohort with 1-year postoperative follow-up included adults undergoing major gastrointestinal and liver surgery across the UK and Ireland between 23 September and 18 November 2015. AKI was defined according to Kidney Disease Improving Global Outcomes (KDIGO) criteria. The primary outcome was death at 1-year after surgery, and the secondary outcome was Major Adverse Kidney Events (MAKE-365). Cox proportionate and multilevel logistic regression were used to account for case mix.

**Results:**

Of 5745 patients across 173 centres, 1-year follow-up data was completed for 3504 patients (62.2 per cent, 126 centres), with attrition largely explained by centre non-participation (63.1 per cent). Some 13.6 per cent (475 of 3504) patients developed AKI by 7 days after surgery (stage 1: 9.2 per cent; stage 2/3: 4.3 per cent). At 1 year, 10.8 per cent (378 patients) experienced a MAKE-365 endpoint (303 patients had died, 61 had renal replacement therapy and 78 had renal dysfunction). Patients who experienced AKI by 7 days after surgery had a higher hazard of death at 1 year for KDIGO stage 1 (hazard ratio 1.50 (95 per cent c.i. 1.08 to 2.08), *P* = 0.016) and KDIGO stage 2/3 (hazard ratio 2.96 (95 per cent c.i. 2.02 to 4.33), *P* < 0.001). Both KDIGO stage 1 (odds ratio 2.09 (95 per cent c.i. 1.50 to 2.92), *P* < 0.001) and stage 2/3 (odds ratio 9.26 (95 per cent c.i. 6.31 to 13.59), *P* < 0.001) AKI were independently associated with MAKE-365.

**Conclusion:**

AKI events within 7 days after gastrointestinal or liver surgery are associated with significantly worse survival and renal outcomes at 1 year.

## Teaser

Acute kidney injury, even stage 1 acute kidney injury, within 7 days after gastrointestinal or liver surgery is associated with significantly worse survival and renal outcomes at 1 year.

## Introduction

Acute kidney injury (AKI) after major abdominal surgery is a common complication^1^, affecting one in seven patients after major abdominal surgery^2^^,^^3^. Early postoperative outcomes associated with AKI have been well documented, with significantly longer inpatient stays, critical care requirement and in-hospital death^4–7^.

The longer-term impact of AKI following major non-cardiac surgery on renal function and death remains unclear. Studies to date have often been limited by the lack of large, prospective, multicentre evidence^2^^,^^8–11^, however they have supported a persistent risk of death associated with postoperative AKI in patients undergoing major surgery, even after the early postoperative period^2^. AKI has also been associated with persistent renal dysfunction and acceleration of progression of chronic kidney disease, but the impact of postoperative AKI and its severity has not been explored previously in prospective series^12–14^.

The aim of this national, prospective cohort study was to describe the incidence of death at 1 year and major adverse kidney events following major gastrointestinal and liver surgery. The secondary aim was to explore the impact of AKI severity on these outcomes.

## Methods

### Study design and setting

A prospective multicentre, observational cohort study (Outcomes After Kidney injury in Surgery (OAKS))^15^, with follow-up up to 1 year after surgery adopted a trainee-research collaborative approach to ‘snapshot’ data collection and management^16^. Adult patients (age 18 years or greater) undergoing elective or emergency gastrointestinal (GI) resection, liver resection or reversal of ileostomy or colostomy, using any operative approach, were eligible for inclusion^3^ (*Table S1*). All centres routinely conducting eligible operations in the UK and Ireland were invited to participate. Consecutive patients were identified by local collaborators across several predefined, 2-week patient-inclusion windows between 23 September and 18 November 2015. Early postoperative outcomes (up to 30 days after surgery) from this cohort have been reported previously^3^. The protocol for this study was prepublished online (www.starsurg.org, publication date: 18 October 2016). An interdisciplinary expert advisory group, with representation from nephrology, critical care, anaesthesiology, surgery and research methodology, provided expert oversight.

To facilitate changes in research team members over the 1-year interval from recruitment to follow-up, patient-identification numbers were stored centrally using a secure REDCap (Research Electronic Data Capture) server^17^, or on a password-protected NHS computer according to local Caldicott Guardian requirement. The full methodology for long-term follow-up in this study has been reported previously^18^. In brief, all participating centres were invited to submit 1-year follow-up data for patients included in the original cohort study. Clinical notes and electronic health records at the site of index admission were reviewed to collect prespecified data, and follow-up was censored at postoperative day 365 with the day of surgery as day 0. All researchers were required to complete online training modules in AKI, case ascertainment, outcome measurement and data governance before beginning data collection.

### Study definitions

The primary outcome was death at 1 year after surgery (all causes). The key secondary outcome was Major Adverse Kidney Events at 1 year after surgery (MAKE-365). This is a validated, composite measure including death (all causes), new requirement for renal replacement therapy (RRT) and/or persistent renal dysfunction (defined as 30 per cent or greater reduction in estimated glomerular filtration rate (eGFR) on last recorded serum creatinine prior to surgery)^19^. Secondary outcomes included the rate of outpatient nephrology review, and the rate and timing of postoperative serum creatinine measurement between discharge and 1 year after surgery.

Postoperative AKI was defined according to creatinine-based Kidney Disease: Improving Global Outcomes (KDIGO) criteria^20^, and severity classified as stage 1 (serum creatinine increase of 26·5–353.5 μmol/l within 48 hours or 1.5 to 1.9-fold from baseline within 7 days) or stage 2–3 (serum creatinine concentration increase of 353.6 μmol/l within 48 hours or 2-fold or greater from baseline within 7 days, or if undergoing unplanned RRT). Postoperative AKI included events occurring within 7 days of the index operation.

### Statistical analysis

There were two anticipated classifications of loss to follow-up in this study. First, it was predicted that 1-year follow-up would not be possible in all participating centres (‘centre non-participation’); for example, due to rotation of trainee research teams away from a participating site, or sites being unable to obtain permission from a local Caldecott Guardian to store linked identifiable data. Second, loss to follow-up was also predicted where sites were unable to collect and/or submit data from specific patients in sites that were able to participate in long-term follow-up (‘patient missing data’). The proportion of missing outcome data at 1 year was described according to these two categories. It was preplanned to explore any impact of attrition bias through comparing patient, disease and operation factors of patients retained within 1-year follow-up *versus* those with no outcome data available. Full exploration of the effects of attrition within this study methodology has been described previously^18^.

Continuous data were plotted to assess for normality, with data formally tested for normality using a Shapiro–Wilk test as required. Normally distributed data were summarized using mean(s.d.) and analysed using the appropriate *t* test. Non-normally distributed data were summarized using median (i.q.r.) and analysed using equivalent tests for non-normal data. Categorical data were cross-tabulated, and differences in proportions were tested using chi-squared or Fisher’s exact test where required. Time-to-event analysis of postoperative creatinine monitoring were censored by death or last known follow-up within 1 year. Statistical analyses were conducted in R, version 3.4.4 (R Foundation for Statistical Computing, Vienna, Austria), with a two-sided significance level of *P* < 0.050 selected *a priori.*

Outcomes were compared across three groups based on the 7-day postoperative AKI status on their index admission (none; stage 1; stage 2/3)^20^. For multivariable analyses, explanatory variables were selected *a priori* based on clinical plausibility in influencing postoperative death or renal outcomes. These included: age, sex, ASA grade, diabetes mellitus (present or absent), baseline eGFR, operative pathology (benign or malignant), operative risk (based on previously described procedure-specific 30-day mortality rates^21^), urgency (elective or emergency) and approach (open or laparoscopic). Finally, site of index admission (hospital) was included in the models as a random effect to account for regional variation.

Differences in survival at 1 year were first assessed using Kaplan–Meier survival curves and log rank tests. Subsequently, Cox proportional hazard models were applied to estimate the hazard of death at any point over the 1 year after surgery. The proportional hazard assumption was evaluated visually via scaled Schoenfeld residual plots and using Schoenfeld global and individual tests. In the event of the assumption being violated, a corresponding time-dependent co-variable was incorporated within an extended Cox regression model^22^. Sensitivity analyses to explore further the long-term relationship between AKI and postoperative death were conducted. One included only patients undergoing elective surgery, and another excluded patients who died in the early postoperative interval (days 0–30) and also incorporated the effect of other major postoperative complications (Clavien–Dindo grade III–IV) up to postoperative day 30^23^. A secondary multilevel, hierarchical binary logistic regression model was constructed to explore the association of postoperative AKI and occurrence of MAKE-365. Effect estimates were presented as adjusted odds ratios with 95 per cent confidence intervals.

### Ethics and reporting

An NHS Health Research Authority tool was completed, that indicated that no formal ethical approval would be required for this study, as it used routinely collected data only, and was anonymized at source before analysis. Exemption from ethics review was confirmed by the South East Scotland Research Ethics Service (*Appendix 1*). UK centres therefore preregistered the study locally as either clinical audit or service evaluation. Local Caldecott Guardian approval was sought for data collection and storage in all centres before study initiation. In the Republic of Ireland, participating centres secured research ethics approval locally, as required by their institutional regulations. These study results are reported in line with STROBE^24^. Contributing authors are recognized in accordance with the consensus guidelines for standardizing reporting of authorship in collaborative research^25^.

## Results

### Baseline characteristics

**Fig. 1:**
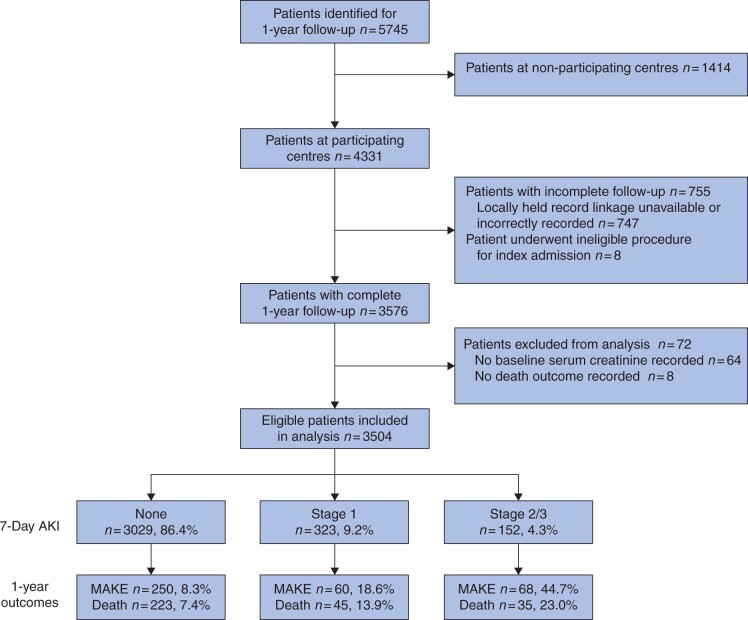
Flow diagram of patient inclusion in 1-year follow-up study from the original Outcomes After Kidney injury in Surgery cohort AKI, acute kidney injury; MAKE, Major Adverse Kidney Events.

Some 5745 patients across 173 centres were included in the original OAKS cohort, and 1-year follow-up was completed for 62.2 per cent (3576 patients) across 126 centres (*Fig. 1*). There were 3504 patients included in the final analysis after exclusion of 72 patients who had no baseline serum creatinine data (64 patients) or had the recorded outcome death at 30 days (8 patients).

### Assessment for attrition bias

The majority of missing outcome data was explained by centre non-participation (63.1 per cent, 1414 of 2241 patients) or issues with local pseudo-anonymized record linkage (33.3 per cent, 747 of 2241 patients). There were few significant differences in attrition with regard to baseline patient characteristics and early postoperative outcomes between groups included and excluded from the analysis (*Table S2*). The rate of open surgery was higher in patients in the included group (62.2 *versus* 52.5 per cent, *P* < 0.001) with a marginally higher rate of malignant pathology (59.1 *versus* 56.2 per cent, *P* = 0.028) and major 30-day postoperative complications (15.9 *versus* 13.9 per cent, *P* = 0.034). However, there was no statistically significant difference in the AKI rate at 7 days between the included and excluded groups (*P* = 0.163).

### Postoperative acute kidney injury

A total of 13.6 per cent of patients (475 of 3504) developed AKI by postoperative day 7, with 9.2 per cent (323 patients) developing stage 1, and 4.3 per cent (152 patients) stage 2/3 AKI. Patients who developed AKI were significantly more likely to be older, male and have a higher co-morbidity burden (including poorer baseline renal function), to undergo higher-risk procedures (emergency or open) and to have experienced major postoperative complications (*Table 1*). There were no significant differences of AKI incidence across type of surgery: upper gastrointestinal (15.2 per cent, 61 of 400 patients), hepatopancreatobiliary (10.9 per cent, 15 of 138 patients) or colorectal (13.5 per cent, 399 of 2966) surgery (*P* = 0.395).

**Table 1:** Baseline demographics of patients with and without postoperative acute kidney injury (AKI)

Characteristic	AKI (*n* = 475)	No AKI (*n* = 3029)	*P*
Age (years)*	67.4(13.4)	62.4(16.2)	<0.001
**Sex**			
Female	175 (11.2)	1388 (88.8)	<0.001
Male	300 (15.5)	1641 (84.5)	
**ASA grade**			
I–II	242 (11.3)	1903 (88.7)	<0.001
III–V	201 (17.9)	923 (82.1)	
Unknown	32 (13.6)	203 (86.4)	
**Diabetes mellitus**			
No	381 (12.7)	2617 (87.3)	<0.001
Yes	94 (18.7)	410 (81.3)	
Baseline eGFR**			
>90	166 (10.9)	1351 (89.1)	<0.001
60 to 89	204 (13.9)	1267 (86.1)	
<60	104 (20.6)	402 (79.4)	
Missing	1 (10.0)	9 (90.0)	
**Operative pathology**			
Benign	186 (13.0)	1244 (87.0)	0.444
Malignant	289 (14.0)	1780 (86.0)	
**Operative urgency**			
Elective	348 (12.8)	2377 (87.2)	0.013
Emergency	127 (16.3)	652 (83.7)	
**Operative approach**			
Minimally invasive	156 (11.8)	1166 (88.2)	0.019
Open	319 (14.7)	1858 (85.3)	
**Operative specialty**			
Colorectal	399 (13.5)	2567 (86.5)	0.395
Upper gastrointestinal	61 (15.2)	339 (84.8)	
Hepatopancreatobiliary	15 (10.9)	123 (89.1)	
**30-day postoperative complication**			
No major	310 (10.5)	2633 (89.5)	<0.001
Major	165 (29.6)	393 (70.4)	

Values in parentheses are percentages unless indicated otherwise. All tests are chi-square tests unless otherwise stated.

*Values are mean(s.d.) with associated 2-sample t-test.

**eGFR on admission (ml/min/1.73 m^2^). AKI, acute kidney injury; ASA, American Society of Anesthesiologists; eGFR, estimated glomerular filtration rate.

### Death at 1 year after surgery

Overall, 8.6 per cent of patients (303 patients) died within 1 year, of whom 31.0 per cent (94 patients) died by day 30, and 69.0 per cent (209 patients) died between days 31 to 365. On univariable survival analysis, developing either stage 1 AKI (hazard ratio 1.95 (95 per cent c.i. 1.42 to 2.69), *P* < 0.001) or stage 2/3 AKI (hazard ratio 3.56 (95 per cent c.i. 2.49 to 5.08), *P* < 0.001) was associated with a higher hazard of death at any point over the first postoperative year (day 0 to 365) (*Table 2*).

**Table 2:** Extended Cox regression model of patient survival from postoperative day 0 to 365 following major gastrointestinal surgery, by AKI stage

	Died (*n* = 303)	Alive (*n* = 3201)	Univariable analysis		**Multivariable analysis** ‡	
			Hazard ratio†	*P*	Hazard ratio†	*P*
**7-day postoperative AKI**						
No	223 (7.4)	2806 (92.6)	–		–	
Stage 1	45 (13.9)	278 (86.1)	1.95 (1.42, 2.69)	<0.001	1.50 (1.08, 2.08)	0.016
Stage 2/3	35 (23.0)	117 (77.0)	3.56 (2.49, 5.08)	<0.001	2.96 (2.02, 4.33)	<0.001
**Age (years)**	71.5(12.4)*	62.3(16.0)*	1.05 (1.04, 1.06)	<0.001	1.04 (1.02, 1.05)	<0.001
**Sex**						
Female	121 (7.7)	1442 (92.3)	–		–	
Male	182 (9.4)	1759 (90.6)	1.22 (0.97, 1.54)	0.085	1.24 (0.97, 1.57)	0.087
**ASA grade**						
I–II	109 (5.1)	2036 (94.9)	–		–	
III–V	172 (15.3)	952 (84.7)	3.23 (2.54, 4.11)	<0.001	1.99 (1.54, 2.57)	<0.001
Unknown	22 (9.4)	213 (90.6)	1.90 (1.20, 3.00)	0.006	1.46 (0.91, 2.33)	0.113
**Diabetes mellitus**						
No	248 (8.3)	2750 (91.7)	–		–	
Yes	55 (10.9)	449 (89.1)	1.33 (1.00, 1.79)	0.053	1.07 (0.79, 1.45)	0.648
Baseline eGFR**						
>90	116 (7.6)	1401 (92.4)	–		–	
60–89	109 (7.4)	1362 (92.6)	0.97 (0.75, 1.26)	0.806	0.75 (0.57, 0.97)	0.032
<60	78 (15.4)	428 (84.6)	2.13 (1.60, 2.84)	<0.001	0.99 (0.72, 1.37)	0.974
**Operative pathology**						
Benign	114 (8.0)	1316 (92.0)	–		–	
Malignant	187 (9.0)	1882 (91.0)	1.12 (0.89, 1.42)	0.333	1.59 (1.19, 2.13)	0.002
**Operative urgency**						
Elective	166 (6.1)	2559 (93.9)	–		–	
Emergency	137 (17.6)	642 (82.4)	3.14 (2.51, 3.94)	0.001	3.30 (2.53, 4.30)	<0.001

Values in parentheses are percentages unless indicated otherwise;

*values are mean(s.d.);

†values in parentheses are 95 per cent confidence intervals.

‡Number in model = 3484, number of groups = 125.

**eGFR on admission (ml/min/1.73 m^2^).

AKI, acute kidney injury; eGFR, estimated glomerular filtration rate.

**Fig. 2:**
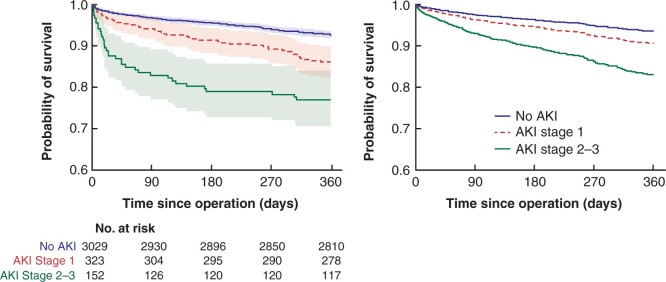
Unadjusted (Kaplan–Meier) and adjusted (extended Cox regression model) survival curves for patient survival from postoperative day 0 to 365, by acute kidney injury (AKI) stage.

On multivariable adjustment, however, the proportional hazards assumption was violated due to a disproportionately higher hazard of death in the 30-day postoperative period (global chi-squared test *P* < 0.001). Therefore, an extended Cox regression model for time-dependent variables was used to account for this (*Fig. 2*, *Table 2*) which stratified postoperative death into early (0–30 days) and intermediate periods (30–365 days). This model demonstrated the hazard of death at any point over the 1-year period was significantly higher in patients with AKI stage 1 (hazard ratio 1.50 (95 per cent c.i. 1.08 to 2.08), *P* = 0.016) and AKI stage 2–3 (hazard ratio 2.96 (95 per cent c.i. 2.02 to 4.33), *P* < 0.001) compared with patients without AKI. This relationship remained consistent in a subgroup analysis of only elective patients (*Table S3*).

**Fig. 3:**
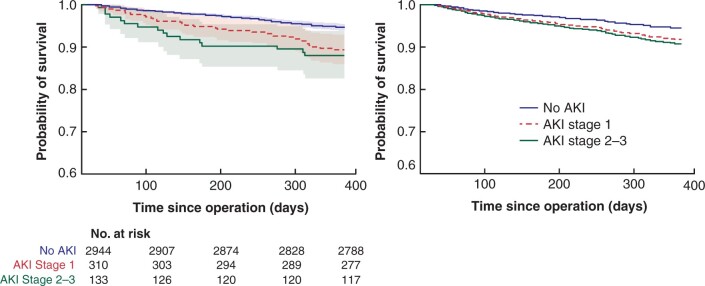
Unadjusted (Kaplan-Meier) and adjusted (Cox regression model) survival curves for patient survival from postoperative day 30 to 365, by acute kidney injury (AKI) stage.

A sensitivity analysis excluding all patients who died within 30 days after surgery was conducted to determine if these differences observed in survival could be attributed to early postoperative deaths alone (*Fig. 3*, *Table 3*). For patients who were alive at postoperative day 30, there was a marginally significant increase in the hazard of death in patients who experienced AKI. This was similar in both stage 1 AKI (hazard ratio 1.50 (95 per cent c.i. 1.01 to 2.21), *P* = 0.042) and stage 2/3 AKI (hazard ratio 1.82 (95 per cent c.i. 1.06 to 3.13), *P* = 0.030).

**Table 3:** Cox regression sensitivity analysis of patient survival from postoperative day 30 to 365 following major gastrointestinal surgery, by AKI stage

	Died (*n* = 209)	Alive (*n* = 3183)	Univariable analysis		Multivariable analysis‡	
			Hazard ratio†	*P*	Hazard ratio†	*P*
**7-day postoperative AKI**						
No	160 (5.4)	2789 (94.6)	–		–	
Stage 1	33 (10.6)	277 (89.4)	2.01 (1.39, 2.93)	<0.001	1.50 (1.01, 2.21)	0.042
Stage 2/3	16 (12.0)	117 (88.0)	2.34 (1.40, 3.91)	0.001	1.82 (1.06, 3.13)	0.030
**Age (years)**	70.8(12.9)*	62.3(16.0)*	1.04 (1.03, 1.05)	<0.001	1.03 (1.02, 1.05)	<0.001
**Sex**						
Female	85 (5.6)	1436 (94.4)	–		–	
Male	124 (6.6)	1747 (93.4)	1.19 (0.91, 1.57)	0.208	1.15 (0.86, 1.52)	0.340
**ASA grade**						
I–II	89 (4.2)	2026 (95.8)	–		–	
III–V	102 (9.7)	948 (90.3)	2.39 (1.80, 3.17)	<0.001	1.48 (1.09, 2.02)	0.012
Unknown	18 (7.9)	209 (92.1)	1.95 (1.17, 3.23)	0.010	1.47 (0.86, 2.50)	0.160
**Diabetes mellitus**						
No	170 (5.9)	2735 (94.1)	–		–	
Yes	39 (8.0)	448 (92.0)	1.38 (0.98, 1.96)	0.068	1.12 (0.78, 1.60)	0.540
Baseline eGFR**						
>90	84 (5.7)	1397 (94.3)	–		–	
60–89	77 (5.4)	1358 (94.6)	0.94 (0.69, 1.29)	0.716	0.71 (0.51, 0.98)	0.037
<60	48 (10.1)	428 (89.9)	1.83 (1.29, 2.61)	0.001	0.94 (0.64, 1.40)	0.780
**Operative pathology**						
Benign	62 (4.5)	1311 (95.5)	–		–	
Malignant	147 (7.3)	1872 (92.7)	1.63 (1.21, 2.19)	0.001	2.20 (1.53, 3.14)	<0.001
**Operative urgency**						
Elective	128 (4.8)	2543 (95.2)	–		–	
Emergency	81 (11.2)	640 (88.8)	2.46 (1.86, 3.24)	<0.001	3.04 (2.18, 4.26)	<0.001
**30-day postoperative complication**						
No major	157 (5.4)	2756 (94.6)	–		–	
Major	52 (10.9)	427 (89.1)	2.11 (1.54, 2.89)	<0.001	1.62 (1.15, 2.27)	0.005

Values in parentheses are percentages unless indicated otherwise;

*values are mean(s.d.);

†values in parentheses are 95 per cent confidence intervals.

‡Number in model = 3392, number of groups = 125.

**eGFR on admission (ml/min/1.73 m^2^).

AKI, acute kidney injury; eGFR, estimated glomerular filtration rate.

### Major adverse kidney events at 1 year

**Fig. 4:**
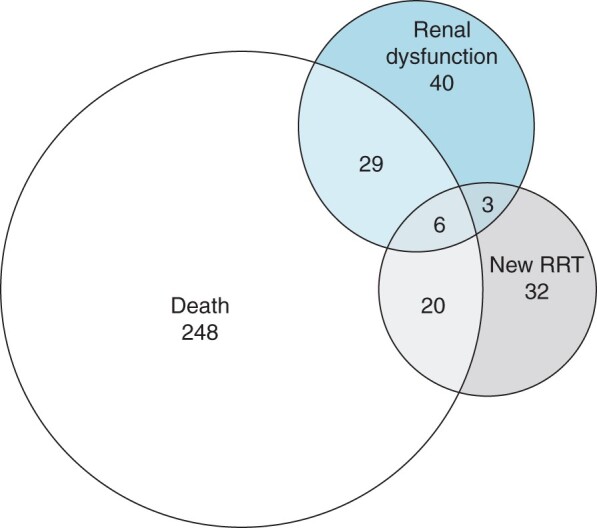
Venn diagram of patients who had a major adverse kidney event (MAKE-365). RRT, renal replacement therapy.

Within the follow-up cohort, there were 378 patients (10.8 per cent) who met the MAKE-365 endpoint (*Fig. 4*). A majority of MAKE-365 outcomes was due to death (303 patients, 80.2 per cent); 61 patients (16.1 per cent) commenced RRT and 78 patients (20.6 per cent) had evidence of persistent renal dysfunction on their last recorded creatinine.

There was a significant association between patients who experienced AKI and the MAKE-365 endpoint on univariable analysis (*Table 4*). Even following multilevel logistic regression, this significantly higher odds of death persisted with a two-fold increase in stage 1 AKI (odds ratio 2.09 (95 per cent c.i. 1.50 to 2.92), *P* < 0.001) and a nine-fold increase in stage 2/3 AKI (odds ratio 9.26 (95 per cent c.i. 6.31 to 13.59), *P* < 0.001), compared with those without AKI.

**Table 4:** Multilevel logistic regression on the occurrence of MAKE-365 outcomes in patients after major gastrointestinal and liver surgery

	MAKE-365 outcomes		Univariable analysis		Multivariable analysis‡	
	Yes (*n* = 378)	No (*n* = 3016)	Odds ratio†	*P*	Odds ratio†	*P*
**7-day postoperative AKI**						
No	250 (8.3)	2779 (91.7)	–		–	
Stage 1	60 (18.6)	263 (81.4)	2.54 (1.85, 3.43)	<0.001	2.09 (1.50, 2.92)	<0.001
Stage 2/3	68 (44.7)	84 (55.3)	9.00 (6.36, 12.70)	<0.001	9.26 (6.31, 13.59)	<0.001
**Age (years)**	69.6(13.4)*	62.3(16.0)*	1.04 (1.03, 1.04)	<0.001	1.03 (1.02, 1.04)	<0.001
**Sex**						
Female	154 (9.9)	1409 (90.1)	–		–	
Male	224 (11.5)	1717 (88.5)	1.19 (0.96, 1.48)	0.110	1.14 (0.90, 1.45)	0.283
**ASA grade**						
I–II	147 (6.9)	1998 (93.1)	–		–	
III–V	203 (18.1)	921 (81.9)	3.00 (2.39, 3.76)	<0.001	1.86 (1.43, 2.42)	<0.001
Unknown	28 (11.9)	207 (88.1)	1.84 (1.18, 2.78)	0.005	1.45 (0.91, 2.32)	0.118
**Diabetes mellitus**						
No	311 (10.4)	2687 (89.6)	–		–	
Yes	67 (13.3)	437 (86.7)	1.32 (0.99, 1.75)	0.051	1.05 (0.77, 1.43)	0.772
**Baseline eGFR** **						
>90	147 (9.7)	1370 (90.3)	–		–	
60–89	139 (9.4)	1332 (90.6)	0.97 (0.76, 1.24)	0.823	0.78 (0.59, 1.02)	0.069
<60	92 (18.2)	414 (81.8)	2.07 (1.56, 2.74)	<0.001	1.06 (0.76, 1.48)	0.737
**Operative pathology**						
Benign	212 (7.8)	2513 (92.2)	–		–	
Malignant	166 (21.3)	613 (78.7)	3.21 (2.57, 4.00)	<0.001	3.35 (2.52, 4.46)	<0.001
**Operative urgency**						
Elective	156 (10.9)	1274 (89.1)	–		–	
Emergency	220 (10.6)	1849 (89.4)	0.97 (0.78–1.21)	0.796	1.41 (1.05, 1.88)	0.020

Values in parentheses are percentages unless indicated otherwise;

*values are mean(s.d.);

†values in parentheses are 95 per cent confidence intervals. AKI, acute kidney injury; eGFR, estimated glomerular filtration rate.

‡Number in model = 3487, number of groups = 126, akaike information criterion (AIC) = 2053.3, C-statistic = 0.788.

**eGFR on admission (ml/min/1.73 m^2^).

### Post-discharge monitoring

**Fig. 5:**
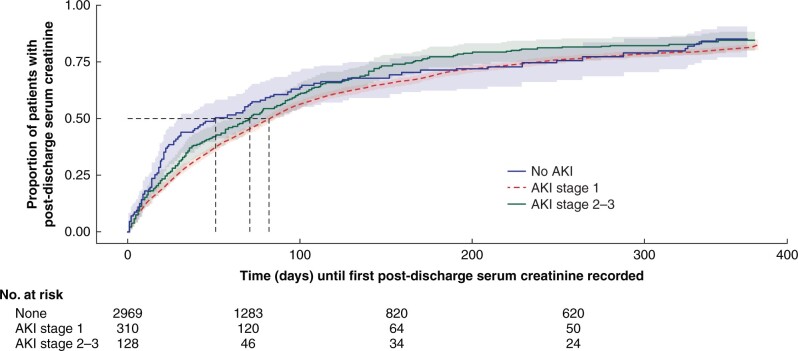
Kaplan–Meier plot of the time until first recorded post-discharge serum creatinine, by acute kidney injury (AKI) stage. Patients censored by death or completion of 1-year follow-up Log rank test *P* = 0.019.

Of the 3411 patients who were alive at 30 days after surgery, 2.4 per cent (81 patients) had a documented outpatient nephrology review within 1 year. There was no significant difference (*P* = 0.163) in the outpatient nephrology review rate for patients who developed AKI stage 1 (3.0 per cent, 9 of 302 patients) or stage 2/3 (0 per cent, 0 of 133 patients), and those who did not (2.4 per cent, 72 of 2967 patients). Similarly, there was no statistically significant difference between those who did and did not develop a MAKE-365 (3.5 *versus* 2.3 per cent, *P* = 0.189). However, there was a significant difference (*P* = 0.019) in the time to first recorded serum creatinine after discharge between AKI groups (*Fig. 5*). There was a median of 50.5 days for patients with AKI stage 2/3 until first serum creatinine recorded after discharge (compared with a median of 69.5 and 80.0 days respectively for patients who experienced AKI stage 1 and no AKI). Overall, one in five patients (80.5 per cent, 664 of 3411 patients) did not receive any follow-up serum creatinine during the follow-up period; 82.9 per cent (257 patients) with stage 1 and 80.2 per cent (105 patients) with stage 2/3 AKI.

## Discussion

AKI is one of the most common complications following major abdominal surgery, yet its long-term implications remain poorly understood. This large, prospective multicentre cohort study across UK and Ireland demonstrates that AKI within 7 days of surgery is associated with higher rates of death, RRT and persistent renal dysfunction at 1 year. Severe effects were seen even following a mild renal insult (KDIGO stage 1), and are sustained over the intermediate postoperative period. Despite these concerns, the authors observed post-discharge monitoring of renal recovery which did not adhere to Royal College of General Practitioners guidance^26^ and low rates of referral to expert nephrology services; enhanced monitoring was not well targeted to individuals at highest risk of deterioration. These data suggest targeted efforts are required to identify better those at risk of postoperative AKI, and determine how best to modify this with enhanced perioperative care and monitoring as part of future research and national quality-improvement efforts.

Data from several previous studies^2^^,^^4^^,^^27^ have reported worse long-term outcomes for patients who develop postoperative AKI after major abdominal surgery. While surgery represents a distinct physiological insult^28^, this association has been reinforced through studies in general inpatient populations^14^^,^^29^. Nevertheless, previous evidence has been limited by retrospective design, risk of bias, small sample sizes, heterogeneity in patient populations and inconsistent definitions. This large prospective study adopted global consensus definitions and timing of AKI^20^ and major renal events^19^, standardized using investigator training and a prepublished study protocol. While cause of death was not collected in this study, previous work has identified complications of cardiovascular disease and malignancy to be among the most common causes identified after AKI following discharge after surgery^30^^,^^31^. The role that AKI has in the causal pathway of deaths remains unclear; AKI may be a marker of physiological frailty, or have a direct effect on risk of death (for example through chronic renal failure or cardiovascular events). Irrespective of the causal role, the authors identify that early postoperative AKI is a key predictor of poor intermediate-term postoperative outcomes. This relationship persisted even in patients undergoing elective surgery, further supporting the significance of AKI. This warrants investigation in future clinical trials on perioperative renoprotective interventions, and consideration of routine implementation of risk stratification for AKI prevention bundles, and enhanced perioperative surveillance^3^^,^^32^.

This study confirms results from previous studies at higher risk of bias^2^^,^^4^^,^^27^^,^^33^ which indicated a sustained increased hazard of death associated with postoperative AKI even after the early postoperative period. Underestimation of the severity of AKI may explain why apparently small increases in creatinine level are significantly associated with adverse outcomes, and why AKI remains associated with ongoing risk even when there is apparent recovery of renal function. Notably, stage 1 and stage 2/3 AKI were associated with similar hazards of death, even after the early postoperative period. This further confirms the importance of recognizing stage 1 AKI in this surgical population, and efforts to mitigate risks of even ‘mild’ AKI where possible. Finally, there was some evidence to suggest that the poorer outcomes associated with chronic kidney disease observed in other studies in non-cardiac surgery^12^^,^^14^^,^^34–36^ may be mediated by the increased likelihood of postoperative AKI. Like a recent prospective study into early clinical outcomes^7^, following multivariable risk adjustment there was no longer an association between preoperative renal function and poorer intermediate clinical outcomes, despite strong univariable associations between these variables.

In this study, there were few changes recorded in post-discharge management following stage 1 or 2/3 AKI. It should be of concern that one in five patients with postoperative AKI did not have a post-discharge serum creatinine recorded and there was no increase in referral for nephrology review after an early postoperative AKI. There was also no significant difference in the time to first serum creatinine measurement after discharge for patients with stage 1 AKI, compared with patients without AKI. This conflicts with Royal College of General Practitioners guidance on post-discharge monitoring of AKI^26^, and may reflect poor recognition of stage 1 AKI, or challenges in communication between secondary and primary care^37^^,^^38^.

While death is clearly an important outcome, a shift toward targeting patient-centred renal outcomes in clinical research has led to the development of the MAKE endpoint^19^. This collaborative research provides useful validation of the stage 2/3 MAKE composite outcome to support its adoption into clinical trials. However, it should be noted that 80 per cent (303 of 378 patients) of MAKE-365 outcomes related to death in this cohort and that this is by definition inclusive of all causes of death (rather than renal-specific causes).

There are several limitations of methodology. First, there was a moderate attrition of patients from recruitment to 1-year follow-up. However, this was largely related to centre non-participation and there was no suggestion of residual attrition bias^18^. The high rate of open surgery included may explain the marginally higher rates of malignant pathology and major 30-day postoperative complications observed compared with the original cohort. However, this was accounted for in multivariable models, and is unlikely to have had a significant effect on the study findings, nor the generalizability of the data; representative patient data from 126 centres were included. Furthermore, it should be acknowledged that the type and stage of any cancers were not accounted for in the analyses and which may be associated with different 1-year survival prognoses. Therefore, while the cohort is considered representative of routine surgical practice at those centres, potential bias may have arisen based on the representation of patients with individual malignant pathologies which may affect generalizability.

Second, this was a pragmatic observational study with no deviation from routine clinical practice. Therefore, it was not feasible to collect all variables that might impact on the incidence of AKI or postoperative death such as frailty^39^ or perioperative haemodynamic instability^40^. Furthermore, data collected were limited to the routinely available data at the index site—an issue noted in other observational studies^7^^,^^41^. Both unmeasured deterioration of renal function and measured deterioration in hospitals without access to community investigations may have led to under-reporting of the primary and secondary outcomes. However, this would reinforce rather than undermine the primary conclusions of this study. Third, it is important to recognize that AKI can often coincide with other early postoperative complications—hence the attempt to adjust for the effects of major complications (not resulting in death) on intermediate-term survival in the sensitivity analysis. While postoperative AKI and resultant organ support (for example dialysis) were determined separately, the Clavien–Dindo grade encompassed these complications. Therefore, bias from adjustment for major 30-day complications in in the 30–365-day period may have reduced the effect of postoperative AKI on death observed. Furthermore, it is possible that minor complications (such as anaemia or bleeding requiring blood transfusion, a Clavien–Dindo grade II complication), which remain unaccounted for in the present model may have residual unmeasured effects on the study outcomes.

## Funding

This study was funded through support from the BJS Society (BJSS). BJSS had no role in study design, data collection, analysis and interpretation, or writing of this report.

*Disclosure*. The authors declare no conflict of interest.

## Supplementary material

Supplementary material is available at *BJS Open* online.

## Supplementary Material

zrab134_Supplementary_Data
