# Supplementary material for: Impact of postoperative acute kidney injury in patients undergoing major gastrointestinal surgery on 1-year survival and renal outcomes: a national multicentre cohort study
Source: BJS Open. 2022 Jan 14;5(6):zrab134. doi: 10.1093/bjsopen/zrab134 (PMC8759520; doi:10.1093/bjsopen/zrab134)
Supplement: zrab134_Supplementary_Data [file zrab134_supplementary_data.docx]

**Supplement**:

Supplementary Table 1: List of eligible OPCS code procedures included in OAKS-1 ^15^

| G01 Oesophagogastrectomy |
| --- |
| G02 Total oesophagectomy |
| G03 Partial oesophagectomy |
| G27 Total gastrectomy |
| G28 Partial gastrectomy |
| G49.1 Gastroduodenectomy |
| G49.2 Total excision of duodenum |
| G49.3 Partial excision of duodenum |
| G58.1 Total jejunectomy |
| G58.4 Partial jejunectomy |
| G69 Ileectomy (resection of ileum) |
| G75.3 Closure of ileostomy |
| H04 Total excision of colon and rectum |
| H04.1 Panproctocolectomy and ileostomy |
| H04.2 Panproctocolectomy and anastomosis of ileum to anus ± creation of pouch |
| H05.1 Total colectomy and anastomosis |
| H05.2 Total colectomy and ileostomy |
| H06.1 Right hemicolectomy and anastomosis |
| H06.4 Right hemicolectomy and ileostomy |
| H08.1 Transverse colectomy and anastomosis |
| H08.4 Transverse colectomy and stoma |
| H06.1 Left hemicolectomy and anastomosis |
| H06.4 Left hemicolectomy and stoma |
| H08.1 Transverse colectomy and anastomosis |
| H08.4 Transverse colectomy and stoma |
| H10.1 Sigmoid colectomy and anastomosis |
| H10.4 Sigmoid colectomy and stoma |
| H15.4 Closure of colostomy |
| H29.1 Subtotal excision of colon and rectum |
| H29.3 Subtotal excision of colon |
| H33.1 Abdominoperineal excision |
| H33.2 Proctectomy |
| H33.3 Anterior resection |
| H33.6 Anterior resection and stoma |
| H33.5 Rectosigmoidectomy and stoma |
| J02.1 Hemihepatectomy |
| J02.3 Resection of segment of liver |
| J02.4 Wedge excision of liver |

**Supplementary Table 2**: Demographics and early (30-day) postoperative outcomes of patients from the original OAKS cohort included and excluded from the current analysis.

|  |  | Included  (n=3504) | Excluded (n=2241) | p |
| --- | --- | --- | --- | --- |
| Age (years) | Mean (SD) | 63.1 (15.9) | 62.9 (16.1) | 0.760 |
| Sex | Female | 1563 (44.6) | 1030 (46.0) | 0.314 |
|  | Male | 1941 (55.4) | 1211 (54.0) |  |
| American Society of Anesthesiologists (ASA) Grade | I-II | 2145 (65.6) | 1373 (67.4) | 0.189 |
|  | III-V | 1124 (34.4) | 665 (32.6) |  |
| Diabetes Mellitus | No | 2998 (85.6) | 1910 (85.5) | 0.875 |
|  | Yes | 504 (14.4) | 325 (14.5) |  |
| Baseline eGFR on admission (ml/min/1.73 m^2^) | >90 | 1517 (43.4) | 970 (44.2) | 0.863 |
|  | 60 to 89 | 1471 (42.1) | 913 (41.6) |  |
|  | <60 | 506 (14.5) | 314 (14.3) |  |
| Operative Pathology | Benign | 1430 (40.9) | 979 (43.8) | 0.028 |
|  | Malignant | 2069 (59.1) | 1256 (56.2) |  |
| Operative Urgency | Elective | 2725 (77.8) | 1760 (78.5) | 0.493 |
|  | Emergency | 779 (22.2) | 481 (21.5) |  |
| Operative Approach | Minimally-invasive | 1322 (37.8) | 1061 (47.5) | <0.001 |
|  | Open | 2177 (62.2) | 1171 (52.5) |  |
| 7-day Postoperative Acute Kidney Injury | No | 3029 (86.4) | 1874 (88.1) | 0.163 |
|  | Stage 1 | 323 (9.2) | 167 (7.9) |  |
|  | Stage 2/3 | 152 (4.3) | 85 (4.0) |  |
| Postoperative Complications (30-day Clavien-Dindo grade) | No major | 2943 (84.1) | 1918 (86.1) | 0.034 |
|  | Major (III-V) | 558 (15.9) | 309 (13.9) |  |
| 30-day postoperative mortality | No | 3410 (97.3) | 2158 (96.9) | 0.264 |
|  | Yes | 94 (2.7) | 69 (3.1) |  |

**Supplementary Table 3**: Cox-regression model of patient survival from postoperative day 0 to 365 following major elective gastrointestinal surgery, by AKI stage.

|  |  | 1-Year Postoperative Mortality | | | |
| --- | --- | --- | --- | --- | --- |
|  |  | Died (n=164) | Alive (n= 2541) | HR (Univariable) | HR (Multilevel) * |
| 7-day Postoperative Acute Kidney Injury | No | 121 (5.1) | 2237 (94.9) | - | - |
|  | Stage 1 | 23 (9.6) | 216 (90.4) | 1.92 (1.23-3.00, p=0.004) | 1.66 (1.05-2.63, p=0.029) |
|  | Stage 2/3 | 20 (18.5) | 88 (81.5) | 4.06 (2.53-6.52, p<0.001) | 3.81 (2.34-6.20, p<0.001) |
| Age (years) | Mean (SD) | 70.2 (11.8) | 62.6 (15.4) | 1.04 (1.03-1.05, p<0.001) | 1.04 (1.02-1.05, p<0.001) |
| Sex | Female | 60 (5.0) | 1129 (95.0) | - | - |
|  | Male | 104 (6.9) | 1412 (93.1) | 1.38 (1.00-1.89, p=0.049) | 1.14 (0.82-1.58, p=0.420) |
| American Society of Anesthesiologists (ASA) Grade | I-II | 75 (4.2) | 1723 (95.8) | - | - |
|  | III-V | 78 (10.6) | 659 (89.4) | 2.64 (1.92-3.62, p<0.001) | 2.06 (1.47-2.88, p<0.001) |
|  | Unknown | 11 (6.5) | 159 (93.5) | 1.57 (0.83-2.96, p=0.161) | 1.42 (0.74-2.73, p=0.290) |
| Diabetes Mellitus | No | 132 (5.8) | 2163 (94.2) | - | - |
|  | Yes | 32 (7.8) | 378 (92.2) | 1.37 (0.93-2.02, p=0.110) | 0.99 (0.67-1.48, p=0.980) |
| Baseline eGFR on admission (ml/min/1.73 m^2^) | >90 | 70 (6.0) | 1094 (94.0) | - | - |
|  | 60to89 | 74 (6.2) | 1124 (93.8) | 1.03 (0.74-1.43, p=0.868) | 0.76 (0.54-1.06, p=0.110) |
|  | <60 | 20 (5.8) | 323 (94.2) | 0.96 (0.59-1.59, p=0.886) | 0.49 (0.29-0.83, p=0.008) |
| Operative Pathology | Benign | 27 (3.2) | 813 (96.8) | - | - |
|  | Malignant | 137 (7.3) | 1728 (92.7) | 2.34 (1.55-3.53, p<0.001) | 1.45 (0.94-2.25, p=0.094) |

Abbreviations: eGFR = estimated Glomerular Filtration Rate. * Number in model = 2705, Number of groups = 125.

Appendix 1: Confirmation of exemption of requirement for ethical review


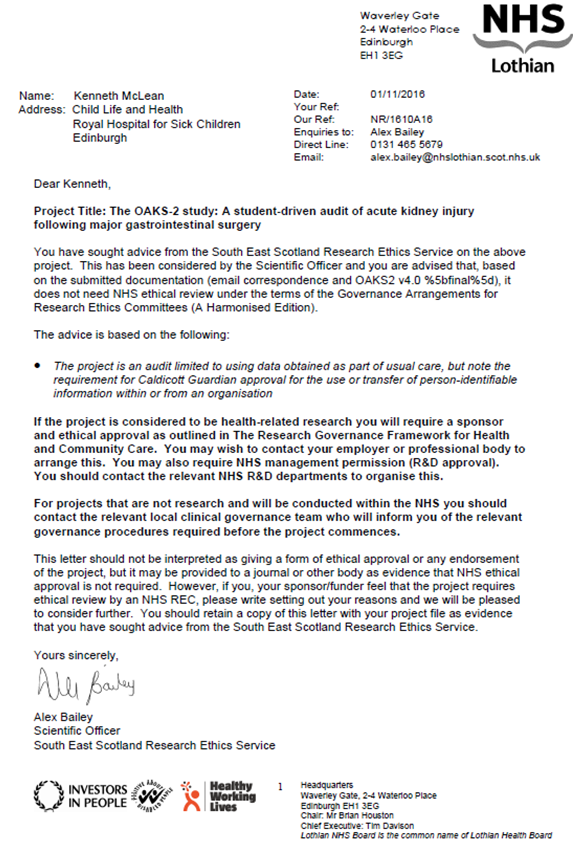


**Appendix 2: Collaborating authors (all PubMed citable)**

**Writing Committee:** Kenneth A McLean *, Sivesh K Kamarajah *, Emily Mills, James C Glasbey, Dmitri Nepogodiev. **denotes joint first authors*

**Data analysis:** Kenneth A McLean.

**Steering Committee:** James C Glasbey*, Aditya Borakati, Joshua Burke, Thomas M Drake, Sivesh K Kamarajah, Kenneth A McLean, Michael F Bath, Henry A Claireaux, Buket Gundogan, Midhun Mohan, Praveena Deekonda, Chia Kong, Holly Joyce, Lisa Mcnamee, Evelina Woin, Chetan Khatri, J Edward Fitzgerald, Ewen M Harrison, Aneel Bhangu, Dmitri Nepogodiev*. **denotes joint senior leads*

**Advisory group:** Nishkantha Arulkumaran, Samira Bell, Fiona Duthie, Jeremy Hughes, Thomas D Pinkney, John Prowle, Toby Richards, Mark Thomas.

**OAKS-1 Regional leads:**

K Dynes (University of Aberdeen, Aberdeen); M Patel, P Patel (Queen Mary University, London); C Wigley, R Suresh (University of Birmingham, Birmingham); A Shaw (University of Bristol, Bristol); S Klimach (Brighton and Sussex Medical School, Brighton); P Jull (University of Cambridge, Cambridge); D Evans, R Preece (Cardiff University, Cardiff); I Ibrahim (University of Dundee, Dundee); V Manikavasagar (Durham University, Durham); R Smith (University of East Anglia); F S Brown (University of Edinburgh, Edinburgh); P Deekonda (Peninsula, Exeter and Plymouth); R Teo, D P Y Sim (University of Glasgow, Glasgow); A Borakati (Hull and York Medical School, Hull and York); A E Logan, I Barai (Imperial College, London); H Amin (Keele University, Keele); S Suresh, R Sethi (King's College, London); W Gul (University of Lancaster); W Bolton (University of Leeds, Leeds); O Corbridge (Leicester Medical School, Leicester); L Horne, M Attalla (University of Liverpool, Liverpool); R Morley, C Robinson (University of Manchester, Manchester); T Hoskins (Newcastle University Medical School, Newcastle upon Tyne); R McAllister (University of Nottingham, Nottingham); S Lee (National University of Ireland, Galway); Y Dennis (University of Oxford, Oxford); G Nixon (Queen’s University Belfast, Belfast); E Heywood (University of Sheffield, Sheffield); H Wilson (Southampton Medical School, Southampton); L Ng, S Samaraweera (St George’s University of London, London); A Mills (Swansea University, Swansea); C Doherty (Trinity College, Dublin); E Woin (University College London, London); J Belchos (University College Dublin, Dublin); V Phan (University of Warwick, Coventry).

**OAKS-2 Regional leads:**

M Arnold (University of Aberdeen, Aberdeen); S Sheik-Ali (Queen Mary University, London); R Suresh (University of Birmingham, Birmingham); A Cordaro (University of Bristol, Bristol); E Mills (Brighton and Sussex Medical School, Brighton); N Lorch (University of Cambridge, Cambridge); D Thomas (Cardiff University, Cardiff); B Ibrahim (University of Dundee, Dundee); S Chee (Dublin); T Ngan (University of East Anglia); S Pronin (University of Edinburgh, Edinburgh); N Thakral (Peninsula, Exeter and Plymouth); T Yeoh (University of Glasgow, Glasgow); J Wilson (Hull and York Medical School, Hull and York); R Goodson (Imperial College, London); P Molloy (Keele University, Keele); M Akhbari (King's College, London); W Gul (University of Lancaster); R Helliwell (University of Leeds, Leeds); S Rees (Leicester Medical School, Leicester); M Al-Attar (University of Liverpool, Liverpool); N Griffiths (University of Manchester, Manchester); J Mayes (Newcastle University Medical School, Newcastle upon Tyne); P Thomas (University of Nottingham, Nottingham); S George (National University of Ireland, Galway); A Thind (University of Oxford, Oxford); M Kerr (Queen’s University Belfast, Belfast); F Shafiq (University of Sheffield, Sheffield); I Yasin (Southampton Medical School, Southampton); M Gallagher (St George’s University of London, London); E Sewart (University College London, London).

**OAKS-1 Collaborators:**

T Chouari, T Gardner, N Goergen, J D B Hayes, C S MacLeod, R McCormack, A McKinley, S McKinstry, W Milligan, L Ooi, N M Rafiq, T Sammut, E Sinclair, M Smith (Aberdeen Royal Infirmary); C Baker, A P R Boulton, J Collins, H C Copley, N Fearnhead, H Fox, T Mah, J McKenna, V Naruka, N Nigam, B Nourallah, S Perera, A Qureshi, S Saggar, L Sun, X Wang, D D Yang (Addenbrooke's Hospital); P Caroll, C Doyle, S Elangovan, A Falamarzi, K Gascon Perai, E Greenan, D Jain, M Lang-Orsini, S Lim, L O'Byrne, P Ridgway, S Van der Laan, J Wong (Adelaide and Meath Hospital, Tallaght); J Arthur, J Barclay, P Bradley, C Edwin, E Finch, E Hayashi, M Hopkins, D Kelly, M Kelly, N McCartan, A Ormrod, A Pakenham (Aintree University Hospital); J Hayward, C Hitchen, A Kishore, T Martins, J Philomen , R Rao, C Rickards (Airedale General Hospital); N Burns, M Copeland, C Durand, A Dyal, A Ghaffar, A Gidwani, M Grant, C Gribbon, A Gruhn, M Leer (Altnagelvin Area Hospital); K Ahmad, G Beattie, M Beatty, G Campbell, G Donaldson, S Graham, D Holmes, S Kanabar, H Liu, C McCann, R Stewart, S Vara (Antrim Area Hospital); O Ajibola-Taylor, E J E Andah, C Ani, N M O Cabdi, G Ito, M Jones, A Komoriyama, P Patel, L Titu (Arrowe Park Hospital); M Basra, P Gallogly, G Harinath, S H Leong, A Pradhan, I Siddiqui, S Zaat (Ashford William Harvey Hospital); A Ali, M Galea, W L Looi, J C K Ng (Ayr Hospital); G Atkin, A Azizi, Z Cargill, Z China, J Elliot, R Jebakumar, J Lam, G Mudalige, C Onyerindu, M Renju, V Shankar Babu (Barnet General Hospital); M Hussain, N Joji, B Lovett, H Mownah (Basildon University Hospital); B Ali, B Cresswell, A K Dhillon, Y S Dupaguntla, C Hungwe, J D Lowe-Zinola, J C H Tsang (Basingstoke and North Hampshire Hospital); K Bevan, C Cardus, A Duggal, S Hossain, M McHugh, M Scott (Bedford Hospital); F Chan, R Evans, E Gurung, B Haughey, B Jacob-Ramsdale, M Kerr, J Lee, E McCann, K O'Boyle, N Reid (Belfast City Hospital); F Hayat, S Hodgson, R Johnston, W Jones, M Khan, T Linn, S Long, P Seetharam, S Shaman, B Smart (Blackpool Victoria Hospital); A Anilkumar, J Davies, J Griffith, B Hughes, Y Islam, D Kidanu, N Mushaini, I Qamar, H Robinson, M Schramm, C Yan Tan (Bradford Royal Infirmary); H Apperley, C Billyard, J M Blazeby, S P Cannon, S Carse, A Göpfert, A Loizidou, J Parkin, E Sanders, S Sharma, G Slade, R Telfer, I Whybrow Huppatz, E Worley (Bristol Royal Infirmary); L Chandramoorthy, C Friend, L Harris, P Jain, M J Karim, K Killington, J McGillicuddy, C Rafferty, N Rahunathan, T Rayne, Y Varathan, N Verma, D Zanichelli (Castle Hill Hospital); M Arneill, F Brown, B Campbell, L Crozier, J Henry, C McCusker, P Prabakaran, R Wilson (Causeway Hospital); U Asif, M Connor, S Dindyal, N Math, A Pagarkar, H Saleem, I Seth, S Sharma, N Standfield, T Swartbol (Charing Cross Hospital); R Adamson, J E Choi, O El Tokhy, W Ho, N R Javaid, M Kelly, A S Mehdi, D Menon, I Plumptre, S Sturrock, J Turner, O Warren (Chelsea and Westminster Hospital); E Crane, B Ferris, C Gadsby, J Smallwood, M Vipond, V Wilson (Cheltenham General Hospital); T Amarnath, A Doshi, C Gregory, K Kandiah, B Powell, H Spoor, C Toh, R Vizor (Chesterfield Royal Hospital); M Common, K Dunleavy, S Harris, C Luo, Z Mesbah, A Prem Kumar, A Redmond, S Skulsky, T Walsh (Connolly Hospital, Blanchardstown); D Daly, L Deery, E Epanomeritakis, M Harty, D Kane, K Khan, R Mackey, J McConville, K McGinnity, G Nixon (Craigavon Area Hospital); A Ang, J Y Kee, E Leung, S Norman, S V Palaniappan, P Partha Sarathy, T Yeoh (Crosshouse Hospital); J Frost, P Hazeldine, L Jones, M Karbowiak, C Macdonald, A Mutarambirwa, A Omotade, M Runkel, G Ryan, N Sawers, C Searle, S Suresh, S Vig (Croydon Hospital); A Ahmad, R McGartland, R Sim, A Song, J Wayman (Cumberland Infirmary); R Brown, L H Chang, K Concannon, C Crilly (Daisy Hill Hospital); T J Arnold, A Burgin, F Cadden, C H Choy, M Coleman, D Lim, J Luk, P Mahankali-Rao, A J Prudence-Taylor, D Ramakrishnan, J Russell (Derriford Hospital); A Fawole, J Gohil, B Green, A Hussain, L McMenamin, L McMenamin, M Tang (Dewsbury Hospital); F Azmi, S Benchetrit, T Cope, A Haque, A Harlinska, R Holdsworth, T Ivo, J Martin, T Nisar, A Patel, K Sasapu, J Trevett, G Vernet (Diana, Princess of Wales Hospital); A Aamir, C Bird, A Durham-Hall, W Gibson, J Hartley, N May, V Maynard (Doncaster Royal Infirmary); S Johnson, C McDonald Wood, M O'Brien, J Orbell, T D Stringfellow, F Tenters, S Tresidder (Dorset County Hospital, Dorchester); W Cheung, A Grant, N Tod (Dr Gray's, Elgin); M Bews-Hair, Z H Lim, S W Lim, M Vella-Baldacchino (Dumfries and Galloway Infirmary); S Auckburally, A Chopada, S Easdon, R Goodson, F McCurdie, M Narouz, A Radford, E Rea, O Taylor, T Yu (Ealing Hospital); M Alfa-Wali, L Amani, I Auluck, P Bruce, J Emberton, R Kumar, N Lagzouli, A Mehta, A Murtaza, M Raja (Epsom Hospital); I S Dennahy, K Frew, A Given, Y Y He, M A Karim, E MacDonald, E McDonald, D McVinnie, S K Ng, A Pettit, D P Y Sim (Forth Valley Royal Hospital); S D Berthaume-Hawkins, R Charnley, K Fenton, D Jones, C Murphy, J Q Ng, R Reehal, H Robinson, S S Seraj, E Shang, A Tonks, P White, A Yeo (Freeman Hospital); P Chong, R Gabriel, N Patel, E Richardson, L Symons (Frimley Park Hospital); D Aubrey-Jones, S Dawood, M Dobrzynska, S Faulkner, H Griffiths, F Mahmood, P Patel, M Perry, A Power, R Simpson (Furness general hospital); A Ali, P Brobbey, A Burrows, P Elder, R Ganyani, C Horseman, P Hurst, H Mann, K Marimuthu, S McBride, E Pilsworth, N Powers, P Stanier (George Eliot Hospital); R Innes, T Kersey (Gilbert Bain Hospital, Shetland); M Kopczynska, N Langasco, N Patel, R Rajagopal (Glan Clwyd Hospital, Rhyl); B Atkins, W Beasley, Z Cheng Lim, A Gill, H Li Ang, H Williams, T Yogeswara (Glangwili General Hospital, Carmarthen); R Carter, M Fam, J Fong, J Latter, M Long, S Mackinnon, C McKenzie, J Osmanska, V Raghuvir, A Shafi, K Tsang, L Walker (Glasgow Royal Infirmary); K Bountra, O Coldicutt, D Fletcher, S Hudson, S Iqbal, T Lopez Bernal, J W B Martin, F Moss-Lawton, J Smallwood, M Vipond (Gloucestershire Royal Hospital); A Cardwell, K Edgerton, J Laws, A Rai, K Robinson, K Waite, J Ward, H Youssef (Good Hope Hospital); C Knight, P Y Koo, A Lazarou, S Stanger, C Thorn, M C Triniman (Great Western Hospital, Swindon); A Botha, L Boyles, S Cumming, S Deepak, A Ezzat, A J Fowler, A M Gwozdz, S F Hussain, S Khan, H Li, B Lu Morrell, J Neville, R Nitiahpapand, O Pickering, H Sagoo, E Sharma, K Welsh (Guys and St.Thomas' Hospital); S Denley, S Khan (Hairmyres Hospital); M Agarwal, N Al-Saadi, R Bhambra, A Gupta, Z A R Jawad, L R Jiao, K Khan, G Mahir, S Singagireson, B L Thoms, B Tseu, R Wei, N Yang (Hammersmith Hospital); N Britton, D Leinhardt, M Mahfooz, A Palkhi, M Price, S Sheikh (Harrogate District Hospital); M Barker, D Bowley, M Cant, U Datta, M Farooqi, A Lee, G Morley, M Naushad Amin, A Parry, S Patel, S Strang, N Yoganayagam (Heartlands Hospital, Birmingham); A Adlan, S Chandramoorthy, Y Choudhary, K Das, M Feldman, B France, R Grace, H Puddy, P Soor (Hereford County Hospital); M Ali, P Dhillon, A Faraj, L Gerard, M Glover, H Imran, S Kim, Y Patrick, J Peto, A Prabhudesai, R Smith, A Tang, N Vadgama (Hillingdon Hospital); R Dhaliwal, T Ecclestone, A Harris, D Ong, D Patel, C Philp, E Stewart, L Wang, E Wong, Y Xu (Hinchingbrooke Hospital); T Ashaye, T Fozard, F Galloway, S Kaptanis, P Mistry, T Nguyen, F Olagbaiye, M Osman, Z Philip, R Rembacken, S Tayeh, K Theodoropoulou (Homerton Hospital); A Herman, J Lau, A Saha, M Trotter (Huddersfield Royal Infirmary); O Adeleye, D Cave, T Gunwa, J Magalhães, S Makwana, R Mason, M Parish, H Regan, P Renwick, G Roberts, D Salekin, C Sivakumar, A Tariq (Hull Royal Infirmary); I Liew, A McDade, D Stewart (Inverclyde Royal Hospital); M Hague, N Hudson-Peacock, C E S Jackson, F James, J Pitt, E Y Walker (Ipswich Hospital); R Aftab, J J Ang, S Anwar, J Battle, E Budd, J Chui, H Crook, P Davies, S Easby, E Hackney, B Ho, S Z Imam, J Rammell (James Cook University Hospital); H Andrews, C Perry, P Schinle (Jersey General Hospital); P Ahmed, T Aquilina, E Balai, M Church, E Cumber, A Curtis, G Davies, Y Dennis, E Dumann, S Greenhalgh, P Kim, S King, K H M Metcalfe, L Passby, N Redgrave, Z Soonawalla, S Waters, A Zornoza (John Radcliffe Hospital and Churchill Hospital); I Gulzar, J Hole, K Hull, H Ishaq, J Karaj, A Kelkar, E Love, S Patel, D Thakrar, M Vine, A Waterman (Kettering General Hospital); N P Dib, N Francis, M Hanson, R Ingleton, K S Sadanand, N Sukirthan (King Georges Hospital, London); S Arnell, M Ball, N Bassam, G Beghal, A Chang, V Dawe, A George, T Huq, A Hussain, B Ikram, L Kanapeckaite, M Khan, D Ramjas, A Rushd, S Sait, M Serry, E Yardimci (King's College Hospital); S Capella, L Chenciner, C Episkopos, E Karam, C McCarthy, W Moore-Kelly, N Watson (King's Mill Hospital); V Ahluwalia, J Barnfield, O Ben-Gal, I Bloom, A Gharatya, K Khodatars, N Merchant, A Moonan, M Moore, K Patel, H Spiers, K Sundaram, J Turner (Kingston Hospital); M F Bath, J Black, H Chadwick, L Huisman, H Ingram, S Khan, L Martin, M Metcalfe, P Sangal, J Seehra, A Thatcher, S Venturini, I Whitcroft (Leicester General Hospital); Z Afzal, S Brown, A Gani, A Gomaa, N Hussein, S Y Oh, N Pazhaniappan, E Sharkey, T Sivagnanasithiyar, C Williams, J Yeung (Leicester Royal Infirmary); L Cruddas, S Gurjar, A Pau, R Prakash, R Randhawa (Luton and Dunstable University Hospital); L Chen, I Eiben, M Naylor, D Osei-Bordom, R Trenear (Maidstone Hospital); J Bannard-Smith, N Griffiths, B Y Patel, F Saeed (Manchester Royal Infirmary); H Abdikadir, M Bennett, R Church, S E Clements, J Court, A Delvi, J Hubert, B Macdonald, F Mansour, R R Patel, R Perris, S Small (Manor Hospital, Walsall); A Betts, N Brown, A Chong, C Croitoru, A Grey, P Hickland, C Ho, D Hollington, L McKie, A R Nelson, H Stewart (Mater Hospital, Belfast); P Eiben, M Nedham (Medway NHS Trust); I Ali, T Brown, S Cumming, C Hunt, C Joyner, C McAlinden, J Roberts, D Rogers, A Thachettu, N Tyson, R Vaughan, N Verma, T Yasin (Morriston Hospital); K Andrew, N Bhamra, S Leong, R Mistry, H Noble, F Rashed, N R Walker, L Watson, M Worsfold, E Yarham (Musgrove Park Hospital, Taunton); H Abdikadir, A Arshad, B Barmayehvar, L Cato, N Chan-lam, V Do, A Leong, Z Sheikh, T Zheleniakova (New Cross Hospital, Wolverhampton); J Coppel, S T Hussain, R Mahmood, R Nourzaie, J Prowle, S Sheik-Ali, A Thomas (Newham Hospital); A Alagappan, R Ashour, H Bains, J Diamond, J Gordon, B Ibrahim, M Khalil, D Mittapalli, Y N Neo, P Patil, F S Peck, N Reza, I Swan, M Whyte (Ninewells Hospital); S Chaudhry, J Hernon, H Khawar, J O'Brien, M Pullinger, K Rothnie, S Ujjal (Norfolk and Norwich University Hospital); S Bhatte, J Curtis, S Green, A Mayer, G Watkinson (North Durham University Hospital); K Chapple, T Hawthorne, M Khaliq, L Majkowski, T A M Malik, K Mclauchlan, B Ng Wei En, T O'Connor, S Parton, S D Robinson, M I Saat, B N Shurovi, K Varatharasasingam, A E Ward (Northern General Hospital); K Behranwala, M Bertelli, J Cohen, F Duff, O Fafemi, R Gupta, M Manimaran, J Mayhew, D Peprah, M H Y Wong (North Middlesex Hospital); N Farmer, C Houghton, N Kandhari, K Khan, D Ladha, J Mayes, F McLennan, P Panahi, H Seehra (Northumbria Specialist Emergency Care Hospital); R Agrawal, I Ahmed, S Ali, F Birkinshaw, M Choudhry, S Gokani, S Harrogate, S Jamal, F Nawrozzadeh, A Swaray, A Szczap, J Warusavitarne (Northwick Park / St. Mark's Hospitals); M Abdalla, N Asemota, R Cullum, M Hartley, C Maxwell-Armstrong, C Mulvenna, J Phillips, A Yule (Nottingham City Hospital); L Ahmed, K D Clement, N Craig, E Elseedawy, D Gorman, L Kane, J Livie, V Livie, E Moss, A Naasan, F Ravi, P Shields, Y Zhu (Perth Royal Infirmary); M Archer, H Cobley, R Dennis, C Downes, B Guevel, E Lamptey, H Murray, A Radhakrishnan, S Saravanabavan, M Sardar, C Shaw, V Tilliridou, R Wright, W Ye (Peterborough City Hospital); N Alturki, R Helliwell, E Jones, D Kelly, S Lambotharan, K Scott, R Sivakumar, L Victor (Pinderfields Hospital); H Boraluwe-Rallage, P Froggatt, S Haynes, Y M A Hung, A Keyte, L Matthews (Poole Hospital); E Evans, P Haray, I John, A Mathivanan, L Morgan, O Oji, C Okorocha, A Rutherford, H Spiers, N Stageman, A Tsui, R Whitham (Prince Charles Hospital, Merthyr); A Amoah-Arko, E Cecil, A Dietrich, H Fitzpatrick, C Guy, J Hair, J Hilton, L Jawad, E McAleer, Z Taylor, J Yap (Princess of Wales, Bridgend); M Akhbari, D Debnath, T Dhir, M Elbuzidi, M Elsaddig, S Glace, H Khawaja, R Koshy, K Lal, L Lobo, A McDermott, J Meredith, M A Qamar, A Vaidya (Princess Royal University Hospital, Orpington); F Acquaah, L Barfi, N Carter, D Gnanappiragasam, C Ji, F Kaminski, S Lawday, K Mackay, S K Sulaiman, R Webb (Queen Alexandra Hospital, Portsmouth); P Ananthavarathan, F Dalal, E Farrar, R Hashemi, M Hossain, J Jiang, M Kiandee, J Lex, L Mason, J H Matthews, E McGeorge, S Modhwadia, T Pinkney, A Radotra, L Rickard, L Rodman, A Sales, K L Tan (Queen Elizabeth Hospital, Birmingham); A Bachi, D S Bajwa, J Battle, L R Brown, A Butler, A Calciu, E Davies, I Gardner, T Girdlestone, O Ikogho, G Keelan, P O'Loughlin, J Tam (Queen Elizabeth Hospital, Gateshead); J Elias, M Ngaage, J Thompson (Queen Elizabeth Hospital, Kings Lynn); S Bristow, E Brock, H Davis, M Pantelidou, A Sathiyakeerthy, K Singh (Queen Elizabeth Hospital, Woolwich); A Chaudhry, G Dickson, P Glen, K Gregoriou, H Hamid, A Mclean, P Mehtaji, G Neophytou, S Potts (Queen Elizabeth University Hospital, Glasgow); D R Belgaid, J Burke, J Durno, N Ghailan, M Hanson, V Henshaw, U R Nazir, I Omar, B J Riley, J Roberts, G Smart, K Van Winsen (Queens Hospital, London); A Bhatti, M Chan, M D'Auria, S Green, C Keshvala, H Li, C Maxwell-Armstrong, M Michaelidou, L Simmonds, C Smith, A Wimalathasan (Queens Medical Centre, Nottingham); J Abbas, C Cairns, Y R Chin, A Connelly, S Moug, A Nair, D Svolkinas (Royal Alexandra Hospital, Paisley); P Coe, D Subar, H Wang, V Zaver (Royal Blackburn Hospital); J Brayley, P Cookson, L Cunningham, A Gaukroger, M Ho, A Hough, J King, D O'Hagan, A Widdison (Royal Cornwall Hospital); R Brown, B Brown, A Chavan, S Francis, L Hare, J Lund, N Malone, B Mavi, A McIlwaine, S Rangarajan (Royal Derby Hospital); N Abuhussein, H S Campbell, J Daniels, I Fitzgerald, S Mansfield, A Pendrill, D Robertson, Y W Smart, T Teng, J Yates (Royal Devon and Exeter Hospital); A Belgaumkar, A Katira, J Kossoff, S Kukran, C Laing, B Mathew, T Mohamed, S Myers, R Novell, B L Phillips, M Thomas, T Turlejski, S Turner, M Varcada, L Warren, W Wynell-Mayow (Royal Free Hospital); R Church, L Linley-Adams, G Osborn, M Saunders, R Spencer, M Srikanthan, S Tailor, A Tullett (Royal Glamorgan Hospital); M Ali, S Al-Masri, G Carr, O Ebhogiaye, S Heng, S Manivannan, J Manley, L E McMillan, C Peat, B Phillips, S Thomas, H Whewell, G Williams (Royal Gwent Hospital, Newport); A Bienias, E A Cope, G R Courquin, L Day, C Garner, A Gimson, C Harris, K Markham, T Moore, T Nadin, C Phillips, S M Subratty (Royal Hampshire Hospital, Winchester); K Brown, J Dada, M Durbacz, T Filipescu, E Harrison, E D Kennedy, E Khoo, D Kremel, I Lyell, S Pronin, R Tummon, C Ventre, L Walls, E Wootton (Royal Infirmary of Edinburgh); A Akhtar, E Davies, D El-Sawy, M Farooq, M Gaddah, H Griffiths, I Katsaiti, N Khadem, K Leong, I Williams (Royal Lancaster Hospital); C S Chean, D Chudek, H Desai, N Ellerby, A Hammad, S Malla, B Murphy, O Oshin, P Popova, S Rana, T Ward (Royal Liverpool University Hospital); T E F Abbott, O Akpenyi, F Edozie, R El Matary, W English, S Jeyabaladevan, C Morgan, V Naidu, K Nicholls, S Peroos, J Prowle, S Sansome, H D Torrance, D Townsend (Royal London Hospital); J Brecher, H Fung, Z Kazmi, P Outlaw, K Pursnani, N Ramanujam, A Razaq, M Sattar, S Sukumar, T S E Tan (Royal Preston Hospital); K Chohan, S Dhuna, T Haq, S Kirby, J Lacy-Colson, P Logan, Q Malik, J McCann, Z Mughal, S Sadiq, I Sharif, C Shingles, A Simon (Royal Shrewsbury Hospital); S Burnage, S S N Chan, A R J Craig, J Duffield, A Dutta, M Eastwood, F Iqbal, F Mahmood, W Mahmood, C Patel, A Qadeer, A Robinson, A Rotundo, A Schade, R D Slade (Royal Stoke University Hospital); M De Freitas, H Kinnersley, E McDowell, S Moens-Lecumberri, J Ramsden, T Rockall, L Wiffen, S Wright (Royal Surrey Hospital, Guildford); C Bruce, V Francois, K Hamdan, C Limb, A J Lunt, L Manley, M Marks, C F E Phillips (Royal Sussex County Hospital); C J F Agnew, C J Barr, N Benons, S J Hart, D Kandage, R Krysztopik, P Mahalingam, J Mock, S Rajendran, M T Stoddart (Royal United Hospital, Bath); B Clements, H Gillespie, S Lee, R McDougall, C Murray, R O'Loane, S Periketi, S Tan (Royal Victoria Hospital, Belfast); R Amoah, R Bhudia, B Dudley, A Gilbert, B Griffiths, H Khan, N McKigney, B Roberts, R Samuel, A Seelarbokus, A Stubbing-Moore, G Thompson, P Williams (Royal Victoria Infirmary, Newcastle); N Ahmed, R Akhtar, E Chandler, I Chappelow, H Gil, T Gower, A Kale, G Lingam, L Rutler, C Sellahewa, A Sheikh, H Stringer, R Taylor (Russell's Hall Hospital); H Aglan, M R Ashraf, S Choo, E Das, J Epstein, R Gentry, D Mills, Y Poolovadoo, N Ward (Salford Royal Hospital); K Bull, A Cole, J Hack, S Khawari, C Lake, T Mandishona, R Perry, S Sleight, S Sultan, T Thornton, S Williams (Salisbury District Hospital); T Arif, A Castle, P Chauhan, R Chesner, T Eilon, S Kamarajah, C Kambasha, L Lock, T Loka, F Mohammad, S Motahariasl, L Roper, S S Sadhra, A Sheikh, T Toma, Q Wadood, J Yip (Sandwell General Hospital); E Ainger, S Busti, L Cunliffe, T Flamini, S Gaffing, C Moorcroft, M Peter, L Simpson, E Stokes, G Stott, J Wilson, J York, A Yousaf (Scarborough General Hospital); A Borakati, M Brown, A Goaman, B Hodgson, A Ijeomah, U Iroegbu, G Kaur, C Lowe, S Mahmood, Z Sattar, P Sen, A Szuman (Scunthorpe General Hospital); N Abbas, M Al-Ausi, N Anto, R Bhome, L Eccles, J Elliott, E J Hughes, A Jones, A S Karunatilleke, J S Knight, C C F Manson, I Mekhail, L Michaels, T M Noton, E Okenyi, T Reeves, I H Yasin (Southampton General Hospital); D A Banfield, R Harris, D Lim, C Mason-Apps, T Roe, J Sandhu, N Shafiq, E Stickler, J P Tam, L M Williams (Southmead Hospital, Bristol); P Ainsworth, Y Boualbanat, C Doull, E Egan, L Evans, K Hassanin, G Ninkovic-Hall, W Odunlami, M Shergill, M Traish (Southport and Formby Hospital); D Cummings (South Tyneside District Hospital, South Shields); S Kershaw, J Ong, F Reid, H Toellner (Stepping Hill Hospital); A Alwandi, M Amer, D George, K Haynes, K Hughes, L Peakall, Y Premakumar, N Punjabi, A Ramwell, H Sawkins (St. Georges Hospital); J Ashwood, A Baker, C Baron, I Bhide, E Blake, C De Cates, R Esmail, H Hosamuddin, J Kapp, N Nguru, M Raja, F Thomson (St. Helier Hospital); H Ahmed, G Aishwarya, R Al-Huneidi, S Ali, R Aziz, D Burke, B Clarke, A Kausar, D Maskill, L Mecia, L Myers, A C D Smith, G Walker, N Wroe (St.James Hospital); C Donohoe, D Gibbons, P Jordan, C Keogh, A Kiely, P Lalor, M McCrohan, C Powell, M Power Foley, J Reynolds, E Silke, O Thorpe, J Tseun Han Kong, C White (St.James University Hospital); Q Ali, J Dalrymple, Y Ge, H Khan, R S Luo, H Paine, B Paraskeva, L Parker, K Pillai, J Salciccioli, S Selvadurai, V Sonagara, L R Springford, L Tan (St. Mary's Hospital, London); S Appleton, N Leadholm, Y Zhang (Stoke Mandeville Hospital); D Ahern, M Cotter, S Cremen, T Durrigan, V Flack, N Hrvacic, H Jones, B Jong, K Keane, P R O'Connell, J O'sullivan, G Pek, S Shirazi (St Vincent's University Hospital); C Barker, A Brown, W Carr, Y Chen, C Guillotte, J Harte, A Kokayi, K Lau, S McFarlane, S Morrison (Sunderland Royal Hospital); J Broad, N Kenefick, D Makanji, V Printz, R Saito, O Thomas (Torbay Hospital); H Breen, S Kirk, C H Kong, A O'Kane (Ulster Hospital); M Eddama, A Engledow, S K Freeman, A Frost, C Goh, G Lee, R Poonawala, A Suri, P Taribagil (University College Hospital); H Brown, S Christie, S Dean, R Gravell, E Haywood, F Holt, E Pilsworth, R Rabiu, H W Roscoe, S Shergill, A Sriram, A Sureshkumar, L C Tan, A Tanna, A Vakharia (University Hospital Coventry and Warwickshire); S Bhullar, S Brannick, E Dunne, M Frere, M Kerin, K Muthu Kumar, T Pratumsuwan, R Quek, M Salman, N Van Den Berg, C Wong (University Hospital Galway); J Ahluwalia, R Bagga, C M Borg, C Calabria, A Draper, M Farwana, H Joyce, A Khan, M Mazza, G Pankin, M S Sait, N Sandhu, N Virani, J Wong, K Woodhams (University Hospital Lewisham); N Croghan, S Ghag, G Hogg, O Ismail, N John, K Nadeem, M Naqi, S M Noe, A Sharma, S Tan (University Hospital of South Manchester); F Begum, R Best, A Collishaw, J Glasbey, D Golding, B Gwilym, P Harrison, T Jackman, N Lewis, Y L Luk, T Porter, S Potluri, M Stechman, S Tate, D Thomas, B Walford (University Hospital of Wales); F Auld, A Bleakley, S Johnston, C Jones, J Khaw, S Milne, S O'Neill, K K R Singh, R Smith, A Swan, N Thorley, S Yalamarthi, Z D Yin (Victoria Hospital, Kirkcaldy); A Ali, V Balian, R Bana, K Clark, C Livesey, G McLachlan, M Mohammad, N Pranesh, C Richards, F Ross, M Sajid (Warrington Hospital); M Brooke, J Francombe, J Gresly, S Hutchinson, K Kerrigan, E Matthews, S Nur, L Parsons, A Sandhu, M Vyas, F White, A Zulkifli, L Zuzarte (Warwick Hospital); A Al-Mousawi, J Arya, S Azam, A Azri Yahaya, K Gill, R Hallan, C Hathaway, I Leptidis, L McDonagh, S Mitrasinovic, N Mushtaq, N Pang, G B Peiris, S Rinkoff (Watford General Hospital); L Chan, E Christopher, M M H Farhan-Alanie, A Gonzalez-Ciscar, C J Graham, H Lim, K A McLean, H M Paterson, A Rogers, C Roy, D Rutherford, F Smith, G Zubikarai (Western General Hospital, Edinburgh); R Al-Khudairi, M Bamford, M Chang, J Cheng, C Hedley, R Joseph, B Mitchell, S Perera, L Rothwell, A Siddiqui, J Smith, K Taylor, O Wroe Wright (West Middlesex University Hospital); H K Baryan, G Boyd, H Conchie, L Cox, J Davies, S Gardner, N Hill, K Krishna, F Lakin, S Scotcher (Weston General Hospital); J Alberts, M Asad, J Barraclough, A Campbell, D Marshall, W Wakeford (West Suffolk Hospital, Bury St Edmunds); P Cronbach, F D'Souza, E Gammeri, J Houlton (Wexham Park Hospital); M Hall, A Kethees, R Patel, M Perera, J Prowle, M Shaid, E Webb (Whipps Cross Hospital); S Beattie, M Chadwick, O El-Taji, S Haddad, M Mann, M Patel, K Popat, L Rimmer, H Riyat, H Smith (Whiston Hospital); C Anandarajah, M Cipparrone, K Desai, C Gao, E T Goh, M Howlader, N Jeffreys, A Karmarkar, G Mathew, H Mukhtar, E Ozcan, A Renukanthan, N Sarens, C Sinha, A Woolley (Whittington Hospital); R Bogle, O Komolafe, F Loo, D Waugh, R Zeng (Wishaw General Hospital); A Crewe, J Mathias, A Mills, A Owen, A Prior, I Saunders (Withybush Hospital); A Baker, L Crilly, J McKeon, H K Ubhi (Wrexham Maelor Hospital); A Adeogun, R Carr, C Davison, S Devalia, A Hayat, R B Karsan, C Osborne, K Scott, C Weegenaar, M Wijeyaratne (Yeovil District Hospital); F Babatunde, E Barnor-Ahiaku, G Beattie, P Chitsabesan, O Dixon, N Hall, N Ilenkovan, T Mackrell, N Nithianandasivam, J Orr, F Palazzo, M Saad, L Sandland-Taylor, J Sherlock (York Hospital); T Ashdown, S Chandler, T Garsaa, J Lloyd, S Y Loh, S Ng, C Perkins, A Powell-Chandler, F Smith, R Underhill (Ysbyty Gwynedd, Bangor).

**OAKS-2 Collaborators:**

N Goergen, A McKinley, C Neary, N Rafiq (Aberdeen Royal Infirmary); A Badran, N Fearnhead, M Leadon, M Yin Lin Ting (Addenbrooke's Hospital); K Conlon, D Ganesan, D O'Connor (Adelaide and Meath Hospital, Tallaght); M J Arthur, Z Panayi, S Rehman (Aintree University Hospital); H Awni, R Rao, A Robinson (Airedale General Hospital); J Baxter, P Loughlin (Altnagelvin Area Hospital); A Ahmed, H Barrow, M T Liviu (Arrowe Park Hospital); G Harinath, S Raveendran, S Sait (Ashford William Harvey Hospital); A Ali, M Latter, S Udalov (Ayr Hospital); M Bergstrom, H Tabry, E West (Barnet General Hospital); S Dindyal, C Gao, H Patel (Basildon University Hospital); M Bath, K Bevan, M Bica (Bedford Hospital); X M Chan, J Lee, S O'Donnell, M Ravindran (Belfast City Hospital); E Blessing, J H De Sousa Magalhaes, P Jain (Castle Hill Hospital); B Campbell, R Evans (Causeway Hospital); S Poo, C Sanghera, N Standfield (Charing Cross Hospital); D Karponis, A Mehdi, R Patel, O Warren (Chelsea and Westminster Hospital); G Boyd, J O'Callaghan, M Vipond (Cheltenham General Hospital); T Amarnath, A Kumar, M Saat (Chesterfield Royal Hospital); S Davidson, A Hylands, E McKie (Craigavon Area Hospital); R Hughes, J Latter, E Leung (Crosshouse Hospital); P Dos Santos Jorge, J Saramunda, S Vig (Croydon Hospital); P Serebriakoff, J Wayman, S K Yen (Cumberland Infirmary); M Coleman, S Leong, I Sajid, T Tolppa (Derriford Hospital); A Fawole, D Kandola, A Khan (Dewsbury Hospital); F Babatunde, A Harlinska, K Sasapu (Diana, Princess of Wales Hospital); A D Durham-Hall, G Fowler, M Glithero (Doncaster Royal Infirmary); J Orbell, T Stringfellow, A Tulloch (Dorset County Hospital, Dorchester); A Bagchi, A Grant, O Onibere (Dr Gray's, Elgin); M Bews-Hair, N Rajaraman (Dumfries and Galloway Infirmary); T Agarwal, S Rabinowicz, A Radford (Ealing Hospital); E Pedlar, A Raja, H Rshaidat (Epsom Hospital); P Y A Aw, E MacKle, E Y L Yap (Forth Valley Royal Hospital); R Charnley, L A M Lim, M Naylor, B Stainer (Freeman Hospital); N Alseed, R Amarasinghe, R Rajagopal (Glan Clwyd Hospital, Rhyl); P Horgan, S Sohrabi, A Wilkinson (Glasgow Royal Infirmary); N Liew, J Smallwood, M Vipond, N Walker (Gloucestershire Royal Hospital); E Mutengesa, T Rankin, K Waite (Good Hope Hospital); E Robertson-Waters, S Stanger, C Thorn (Great Western Hospital, Swindon); A Botha, A Fowler, T Suri, P Vickers (Guys and St.Thomas' Hospital); S Denley, W Johnston (Hairmyres Hospital); L Jiao, A Pain, K Vutipongsatorn (Hammersmith Hospital); A Kale, R S Karri, K Waite (Heartlands Hospital, Birmingham); C Johnson, J Smith, C Walsh (Hillingdon Hospital); N Dewan, J Prowle, K Theodoropoulou (Homerton Hospital); P Jain, T Nisar (Hull Royal Infirmary); A Ali, L Chung, J Thomas (Inverclyde Royal Hospital); M Abbas, S Mookerjee, J Pitt (Ipswich Hospital); E Budd, T Fung, M Li, D MacAfee (James Cook University Hospital); N Havers, A Kelkar (Kettering General Hospital); M Hanson, R Ingleton, N Sukirthan (King Georges Hospital, London); A Chang, I Eiben, M Qamar (King's College Hospital); H Javanmard, N Watson (King's Mill Hospital); D Bahadori, I Bloom, G Pike (Kingston Hospital); J Black, M Metcalfe, A Radhakrishnan, J Seehra (Leicester General Hospital); K Almeida, H Amin, R Holdsworth, J Yeung (Leicester Royal Infirmary); S Gurjar, R Jones, M Patel (Luton and Dunstable University Hospital); A Alam, H Ali (Maidstone Hospital); J BannardSmith, R Khaw, A Rais (Manchester Royal Infirmary); R Ahluwalia, E Briggs, H Gil (Manor Hospital, Walsall); J Clements, R Cowden, L McCarthy (Mater Hospital, Belfast); N Bassam, S Chan, S F Hussain (Medway NHS Trust); R Hryniv, H Noble, J Olivier (Musgrove Park Hospital, Taunton); J Coppel, J Prowle, S Sait (Newham Hospital); E Elseedawy, A Hassane, I Ibrahim, T Melaugh, (Ninewells Hospital); A Ali, L Ashraf, S Green (North Durham University Hospital); K Chapple, E Heywood, N Ngonyamo, I Nyamali, A Patil (Northern General Hospital); T Bamford, O Fafemi, C Grieco (North Middlesex Hospital); K Khan, A Martin, H Seehra (Northumbria Specialist Emergency Care Hospital); A Burke-Smith, N Johnson, G Samarth, K Sun, J Warusavitarne (Northwick Park / St. Mark's Hospitals); S Green, C Maxwell-Armstrong, J Sivaraj (Nottingham City Hospital); A Campbell, M Elseedawy, E Elseedawy, O Kouli (Perth Royal Infirmary); S Bradbury, R Dennis, H Walji (Peterborough City Hospital); J Hale, P Haray (Prince Charles Hospital, Merthyr); P Eiben, A Light, T Singhal (Princess Royal University Hospital, Orpington); N Carter, F Ewbank, C Perrott (Queen Alexandra Hospital, Portsmouth); I Chappelow, R Hashemi, A Lee, J Matthews, T Pinkney (Queen Elizabeth Hospital, Birmingham); M Byrne, H Eltyeb, P O'Loughlin (Queen Elizabeth Hospital, Gateshead); C Donaldson, O Oke (Queen Elizabeth Hospital, Woolwich); K Bisset, P Glen, S Norman, L Tan (Queen Elizabeth University Hospital, Glasgow); M Ahmed, C Maxwell-Armstrong, S Rangarajan, J Sivaraj (Queens Medical Centre, Nottingham); C Hancock, S Moug, S Smith (Royal Alexandra Hospital, Paisley); G Nowell, B Rigney, A Widdinson (Royal Cornwall Hospital); C Boereboom, J Lund, W Simpson, J Wright (Royal Derby Hospital); I Fitzgerald, S Mansfield, E Shakweh, K Whitehurst (Royal Devon and Exeter Hospital); Z Lee, B Pinnell, G Williams (Royal Gwent Hospital, Newport); R Broll, T Drake, E Harrison, C McCann (Royal Infirmary of Edinburgh); T Abbott, S Mahdi, F Nawab, J Prowle (Royal London Hospital); B Butcher, P D Loganathan, L A Paterson, K Pursnani (Royal Preston Hospital); J Atley, K Hamdan, E Mills (Royal Sussex County Hospital); B Clements, G Donaldson, L Eaton (Royal Victoria Hospital, Belfast); R Aftab, M Gough, B Griffiths, C Ng, G Nolan (Royal Victoria Infirmary, Newcastle); J Archer, V Do, S Sharma (Russell's Hall Hospital); J Epstein, P Sodde, B J Storey (Salford Royal Hospital); H Ahmad, N Akram, T Sami, F Sheldon (Sandwell General Hospital); H Croft, L Han, K Lasithiotakis (Scarborough General Hospital); J Acharya, O Adeleye, G Kaur (Scunthorpe General Hospital); N Dabab, P Kangesu, J Knight, K Srikathrikamanathan, H Wilson (Southampton General Hospital); E Dell, L Ellis, K McDonald, D Sobhanpanah (Southmead Hospital, Bristol); K Foster, J Mogg, S Subramonia (South Tyneside District Hospital, South Shields); P Hill, A Rahem, F Reid (Stepping Hill Hospital); R Bachar, N Greenough, L Hlukha, A Ramwell (St. Georges Hospital); S Carlton-Carew, M Murray, A Raja (St. Helier Hospital); D Burke, M El-Haddad, L B Mecia, N Patel (St.James Hospital); R Bhatt, W J Koay, L Y H Low, J Reynolds (St James University Hospital); S Abbott, H Devan Nair, J J Lee, R O'Connell (St Vincent's University Hospital); W Carr, S Davies, S Unsworth (Sunderland Royal Hospital); J Ashcroft, D Lazenby, D Subar (The Royal Blackburn Hospital); S Choi, S Rinkoff, N Sarens, M Varcada (The Royal Free Hospital); N Ellerby, A Hammad, N McCartan, U Muhammad (The Royal Liverpool University Hospital); M Howlader, E Norman, P Polly (The Whittington Hospital); S Brown, T Clark, N Thakral (Torbay Hospital); P Hann, R Henderson, S Kirk (Ulster Hospital); S Gupta, T Richards, J Ting (University College Hospital); M Byrne, C Byrne, J Cheema, S Walsh (University Hospital Galway); C Borg, J Hardie, Y Sardar (University Hospital Lewisham); B Hughes, S Saeed, F Saeed, A Sharma (University Hospital of South Manchester); E Ang, B Kansu, M Stechman, R Walford, C Woodward (University Hospital of Wales); S Adeyemi, R Awad, L Imam, I Leptidis (Watford General Hospital); E D Kennedy, H Patterson, Z M Soh, L Walls, J D Yau (Western General Hospital, Edinburgh); B Ali, D Evans, J Smith (West Middlesex University Hospital); E James, V E Kantola, K Krishna (Weston General Hospital); H Naeem, J Prowle (Whipps Cross Hospital); O Komolafe, E Tilling (Wishaw General Hospital); C Osborne, J Schuster Bruce, C Weegenaar (Yeovil District Hospital); P Chitsabesan, A Goaman, C Goode, N Nithianandavisam (York Hospital)
